# Bonding Nature of B_2_L_2_‑SiR_2_ (L = NHC, Silylene; R = H, Cl): Metallomimetic, Silylene, or Diboryne?

**DOI:** 10.1021/acs.inorgchem.6c02220

**Published:** 2026-08-12

**Authors:** Huaiyu Zhang, Rui Ma, Huixuan Sun, Jinshuai Song, Yandong Duan, Yirong Mo

**Affiliations:** † Institute of Computational Quantum Chemistry, and Hebei Key Laboratory of Inorganic Nanomaterials, College of Chemistry and Materials Science, 66447 Hebei Normal University, Shijiazhuang 050024, China; ‡ Green Catalysis Center, and College of Chemistry, 12636 Zhengzhou University, Zhengzhou 450001, China; § Hebei Key Laboratory of Photoelectric Control on Surface and Interface, School of Sciences, 83524 Hebei University of Science and Technology, Shijiazhuang 050018, China; ∥ Department of Nanoscience, Joint School of Nanoscience and Nanoengineering, 14616 University of North Carolina at Greensboro, Greensboro, North Carolina 27401, United States

## Abstract

Low-valent main-group compounds represent a highly active frontier field, owing to their unique bonding characteristics and transition-metal-like reactivity in activating inert small molecules. As prototypical systems, diborynes and silylenes have attracted considerable attention, yet the fundamental nature of their bonding remains largely elusive. Herein, we report a systematic theoretical study of the bonding nature and substituent effects in B_2_L_2_-SiR_2_ complexes (L = NHC, silylene; R = H, Cl) using density functional theory (DFT) and the block-localized wave function energy decomposition (BLW-ED) method at the M06-2X-D3/6-311+G­(d) level. Our calculations reveal that SiH_2_ undergoes a HOMO–LUMO inversion induced by Pauli repulsion upon approaching diborynes, thereby enabling a synergistic Dewar–Chatt–Duncanson (DCD)-type orbital interaction. In this mode, it is the silylene that behaves like a metallomimetic center with π_//_-back-donation as the dominant charge-transfer contributor. By comparison, transition-metal silylene complexes are governed by electrostatic attraction, and SiH_2_ simply acts as a classical σ-donor ligand with negligible π-back-donation. Chlorine substitution imposes divergent effects on the two types of interactions. These findings unveil the bonding behavior of silylenes and advance the fundamental understanding of metallomimetic chemistry in low-valent main-group systems, offering valuable theoretical insights for the rational design of novel main-group complexes.

## Graphical abstract

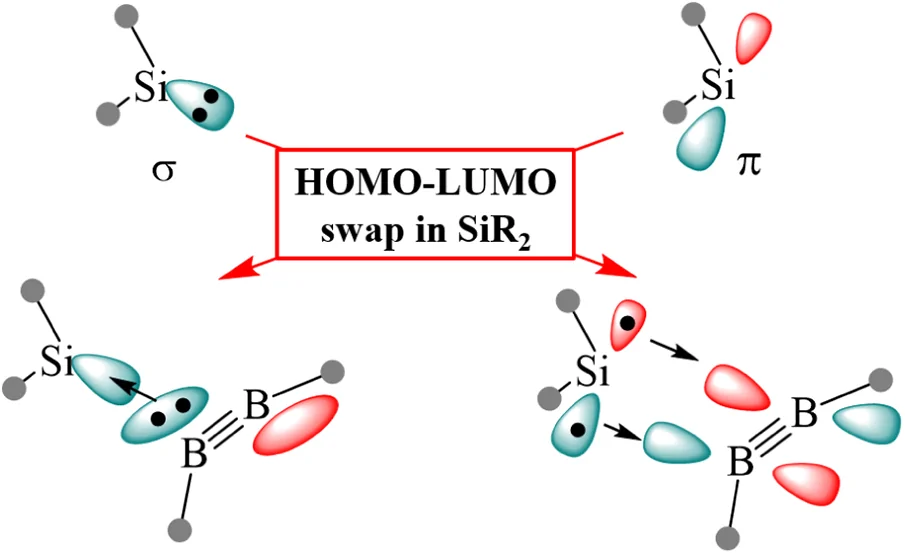

## Introduction

Low-valent main-group compounds represent a vibrant research frontier in inorganic and organometallic chemistry, owing to their unusual electronic structures, distinctive bonding characteristics, and fascinating transition-metal-like reactivity in small-molecule activations.^1–5^ Among these systems, species bearing boron–boron multiple bonds, particularly diborynes L → B≡B ← L (L = Lewis base ligand), form a unique family of multiply bonded compounds whose intrinsic properties differ greatly from their carbon-based alkyne analogues.^3,6–14^

Historically, diborynes were considered highly reactive transient intermediates that could only be detected under extreme cryogenic conditions. In 2002, Zhou and coworkers first characterized the carbonyl-stabilized diboryne analogue OCB≡BCO (Figure 1a) using matrix-isolation infrared spectroscopy, delivering the first experimental proof of B≡B triple-bond character.^6^ A landmark breakthrough came in 2012, when Braunschweig et al. isolated the first room-temperature-stable N-heterocyclic carbene (NHC)-stabilized diboryne (Figure 1b, B_2_(IDip)_2_), unambiguously confirming the existence of a genuine B≡B triple bond.^7,8^ Shortly afterward, the same group substituted IDip ligands with CAACs,^11^ SIDips^12^ and IDeps^14^ (Figure 1b), affording a new family of diborynes with tunable electronic and reactivity properties.

**Figure 1:**
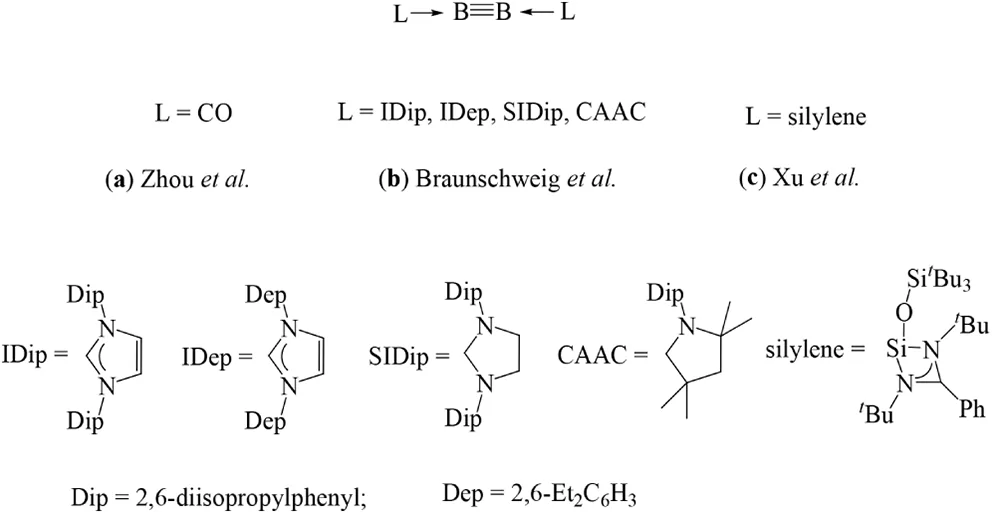
Examples of diborynes featuring B≡B multiple bonds in the literature: (**a**) with the ligand CO; (**b**) with ligands IDips, CAACs, SIDips, and IDeps; (**c**) with amidinato silylene ligands.

In terms of reactivity, diborynes exhibit remarkable transition-metal-mimetic behavior in π-coordination and the activation of inert small molecules including CO and H_2_.^9,10,12,15^ In 2018, our group utilized the block-localized wave function (BLW) method,^16–19^ a rigorous ab initio valence bond (VB) theory variant, to explore the mechanism of CO activation by B_2_(NHC)_2_.^20^ Calculations show that strong Pauli repulsion between the carbon lone pair of CO and one of the π bonds in diboryne triggers spontaneous HOMO–LUMO swap in B_2_(NHC)_2_ without photoexcitation. This orbital rearrangement ultimately enables both the “*in-situ*” HOMO and HOMO–1 of the diboryne to interact simultaneously with the two π* antibonding orbitals of CO, establishing effective back-donation and giving rise to substantial CO activation via the Dewar–Chatt–Duncanson^21,22^ -type mechanism analogous to transition-metal centers. More recently, Xu et al. reported that amidinato silylene ligands can efficiently stabilize diborynes (Figure 1c), which are capable of undergoing cycloaddition with silylenes to construct aromatic boron-containing heterocycles.^23^

As heavier analogues of carbenes, silylenes possess a divalent Si­(II) center featuring one lone pair and an empty 3*p* orbital. Benefiting from the unique electronic structure, silylenes serve as versatile tunable ligands for stabilizing low-valent species and constructing functional metal complexes in main-group and coordination chemistry.^5,24–31^ Among these systems, transition-metal silylene complexes display excellent catalytic performance and have emerged as a thriving research frontier in recent years.^25–32^ In conventional transition-metal silylene complexes, silylenes typically act as σ-donors and π-acceptors.^25,28,32,33^ The dominant σ-donation from the silylene to the metal center affords a planar silicon geometry, while extensive π-back-donation from the metal into the vacant *p* orbital of silicon induces significant pyramidalization at the silicon center.^25,28,32,33^ In contrast, the silicon–carbonyl complexes recently independently reported by Schulz et al.^34^ and Inoue et al.,^35^ feature silylenes with remarkable metallomimetic character that function as σ-acceptors instead.

This raises an intriguing question: when diborynes and silylenes interact, which fragment will display transition-metal-mimetic character within the resulting adduct? If diborynes serve as metallomimetic centers, silylenes may behave analogously to CO in diboryne-CO complexes, triggering HOMO–LUMO inversion at the B_2_ unit and donating σ-electrons toward the diboryne. Alternatively, if silylenes possess transition-metal-like properties and act as σ-acceptors, the underlying bonding mode between silylenes and diborynes remains unclear. To resolve this mechanistic ambiguity, we present a systematic theoretical analysis of the bonding nature in B_2_L_2_-SiR_2_ complexes (L = NHC, silylene; R = H, Cl). The work aims to deepen the fundamental understanding of low-valent boron and silicon chemistry and provide new insights into the metallomimetic chemistry of main-group compounds.

## Theoretical Methods and Computational Details

Two major theoretical frameworks, molecular orbital (MO) theory and VB theory, underpin modern quantum-mechanical descriptions of molecular systems. Approaches within both frameworks differ fundamentally in their orbital construction. MO theory adopts fully delocalized, and orthogonal orbitals, whereas VB methods employ localized and nonorthogonal orbitals rooted in classical bonding concepts.^36,37^ VB theory excels in providing intuitive pictorial descriptions of chemical bonding, while MO theory delivers superior computational efficiency. To combine the advantages of both MO and VB theories, we developed the BLW method.^17–19^ In the BLW method, all electrons and primitive basis functions are partitioned into several subgroups (blocks). Orbitals are fully localized within each block, orthogonal to other orbitals in the same block in the manner of MO theory, and nonorthogonal to orbitals in different blocks, just as in VB theory. The BLW method is the simplest variant of ab initio VB theory and is available at the DFT level. Such strict orbital localization allows quantitative evaluation of electron delocalization impacts on molecular stability, geometry, electronic structure, and spectral and other properties.

The energy decomposition scheme based on the BLW method (BLW-ED) partitions the total binding energy into several physically interpretable components.^38,39^ For B_2_L_2_-SiR_2_ complexes, the binding energy (Δ*E*_b_) between diborynes and silylene is composed of two parts, namely, the deformation energy (Δ*E*_def_) from the optimized free monomer structures to the distorted geometries in the complex structure, and the interaction energy (Δ*E*_int_) between deformed monomers as

1 $$\Delta E_{b}=\Delta E_{\mathrm{def}}+\Delta E_{\mathrm{int}}$$

The interaction energy is further decomposed into three key terms as

2 $$\Delta E_{\mathrm{int}}=\Delta E_{\mathrm{steric}}+\Delta E_{\mathrm{pol}}+\Delta E_{\mathrm{CT}}$$

where Δ*E*_steric_ includes electrostatic interactions, Pauli exchange repulsion, and electron correlation if the computations are run at the DFT level, Δ*E*_pol_ describes the energetic stabilization arising from the electron density relaxation within individual monomers in response to mutual electrostatic fields, and Δ*E*_CT_ represents the charge-transfer stabilization energy arising from the penetration of electrons among monomers after the basis set superposition error (BSSE) correction.^40^

A variety of alternative energy decomposition analysis (EDA) methods, including ETS-NOCV,^41,42^ SAPT,^43^ and GKS-EDA,^44,45^ are also available to quantify the individual contributions of Pauli repulsion, electrostatics, polarization, and orbital interactions to the total interaction energy. Nevertheless, all EDA components are not experimentally observable quantities. Except for electrostatic interactions, the physical implications of remaining EDA terms vary across different methods, as their definitions rely on algorithmic formulations and orbital localization strategies. Notably, these methods fail to trace the dynamic evolution of orbitals during the formation of chemical bonds. In contrast, the BLW method possesses unique advantages of strictly localized orbitals and self-consistent optimization. It enables the clear characterization of both localized (diabatic) and delocalized (adiabatic) electronic states, allowing precise separation of σ- and π-electron delocalization effects. Furthermore, the “*in situ*” orbital analysis approach^20,46,47^ derived from the BLW method can trace the evolution of individual orbital interactions, uncover the intrinsic mechanism of chemical bonding, and provide physically intuitive bonding pictures for fundamental chemical interpretation.

Experimentally known diboryne compounds are exclusively stabilized by N-heterocyclic carbenes^7,11,12,14^ and amidinato silylenes.^23^ Herein, we computationally examined these two experimentally relevant ligand-stabilized diboryne species. Consistent with our previous work on the diboryne-CO interaction,^20^ all substituents were replaced with hydrogen atoms to simplify the model for the NHC ligand. For the silylene ligands reported by Xu et al.,^23^ the steric bulk of peripheral substituents significantly affects the geometry of B_2_L_2_-SiR_2_ (see Figure S1 in Supporting Information). Accordingly, butyl groups on nitrogen, silyl groups on oxygen, and methyl groups on the heterocyclic carbon atoms were retained in the initial model. To reduce computational cost while preserving key electronic features, all remaining heterocyclic substituents were replaced with hydrogen atoms, and only the positions of H atoms were optimized in the final model to analyze the interaction between diboryne (B_2_L_2_) and silylene (SiR_2_).

Throughout this work, all investigated molecules were treated in the singlet electronic state. Geometry optimizations were performed using Gaussian 16 ^48^ at the DFT (M06-2X-D3)^49^ level with the 6-311+G­(d)^50–52^ basis sets for main-group elements and the SBKJC^53–55^ basis sets for transition-metals. The rationality of truncated substituents, basis sets and density functionals has been validated, with corresponding data provided in the Supporting Information. Harmonic vibrational calculations were performed to assess the nature of the stationary points on the potential energy surfaces. Unless otherwise noted, subsequent single-point energy calculations with conventional DFT and BLW methods were carried out at the same level using the GAMESS program^56^ and our in-house BLW code, which is ported to GAMESS. Natural Bond Orbital (NBO) analyses^57–59^ were performed using the NBO 6 program.^60^

## Results and Discussion

### Geometries and Electronic Structures of B_2_L_2_ (L = NHC, Silylene)

The optimized geometries of free Lewis-base-stabilized diborynes B_2_L_2_ (L = NHC, silylene) are shown in Figure 2. The B–B bond length is determined to be 1.444 Å for B_2_(NHC)_2_ and 1.449 Å for B_2_(Sil)_2_ (silylene-stabilized diboryne). These computed values are consistent with both of typical NHC-stabilized diboryne species^7,20^ and the experimental results reported by Xu et al.^23^ Figure S2 presents three natural bond orbitals localized between the two boron atoms in both NHC and silylene stabilized diborynes, comprising one σ bond and two π bonds. It confirms that both NHC and silylene ligands effectively stabilize the highly electron-deficient B_2_ core while preserving the intrinsic triple-bond character. The Wiberg bond indices of the B–B bond in B_2_(NHC)_2_ and B_2_(Sil)_2_ are 2.173 and 2.505, respectively. The lower bond index in B_2_(NHC)_2_ agrees with our previous results and is attributed to π electron back-donation from the central B_2_ moiety to NHC ligands. For B_2_(Sil)_2_, the silylene ligand donates electron density to the B–B π* antibonding orbital only via hyperconjugation, leading to a slightly larger Wiberg bond index.

**Figure 2:**
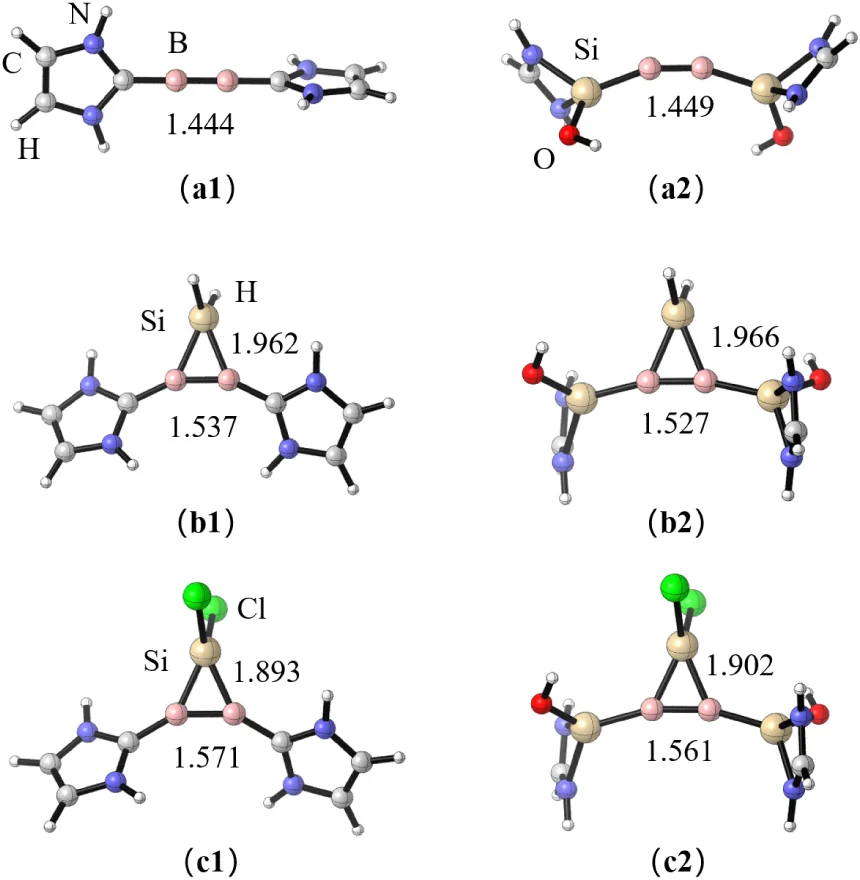
Optimized structures of **(a1)** B_2_(NHC)_2_, **(a2)** B_2_(Sil)_2_, **(b1)** B_2_(NHC)_2_-SiH_2_, **(b2)** B_2_(Sil)_2_-SiH_2_, **(c1)** B_2_(NHC)_2_-SiCl_2_ and **(c2)** B_2_(Sil)_2_-SiCl_2_ with B–B and B–Si bond lengths (Å) computed at the M06-2X-D3/6-311+G­(d) level.

### Bonding Nature of B_2_L_2_-SiH_2_ (L = NHC, Silylene)

Upon coordination with SiH_2_ to form three-membered B–B–Si heterocyclic adducts, the B_2_ core of diboryne aligns perpendicular to SiH_2_ with elongated B–B bonds. Specifically, the B–B bond length increases to 1.537 Å for B_2_(NHC)_2_-SiH_2_ and 1.527 Å for B_2_(Sil)_2_-SiH_2_, with B–B Wiberg indices decreasing to 1.578 and 1.731, far below the values in the diboryne monomers. At the same time, the B–Si bond indices are 0.947 and 0.899, respectively. Consistent with the NBO orbital features presented in Figure 3, these results confirm the formation of a three-membered B–B–Si ring in both adducts. In other words, the central B_2_ moiety breaks one π bond to form two σ bonds with SiH_2_, resulting in tetrahedrally bonded silicon. Notably, both complexes exhibit highly similar NBO orbital profiles and comparable binding energies (Δ*E*_b_, −77.0 and −81.6 kcal/mol, respectively). Thus, NHC and silylene ligands therefore barely alter B_2_L_2_-SiH_2_ (L = NHC, silylene) interactions. We hereafter focus on B_2_(NHC)_2_-SiH_2_ to explore the intrinsic bonding mechanism.

**Figure 3:**
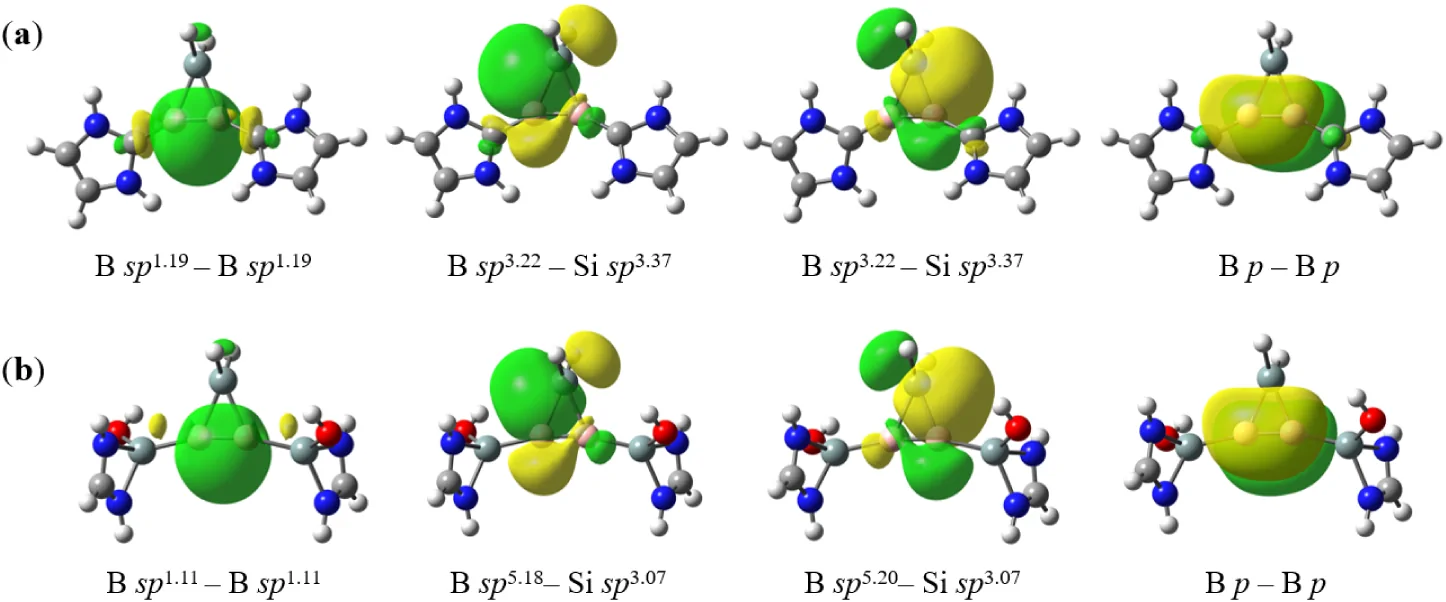
NBOs of (a) B_2_(NHC)_2_-SiH_2_ and (b) B_2_(Sil)_2_-SiH_2_.

We performed detailed energy decomposition analysis using the BLW-ED method, with all energy components systematically summarized in Table 1. The deformation energy (18.3 kcal/mol) is close to that in the B_2_(NHC)_2_-CO system and arises mainly from the structural distortion of B_2_(NHC)_2_, whose optimized geometry has D_2d_ symmetry.

**Table 1:** Computed Energy Components (kcal/mol) with the BLW-ED Approach

Complex	Δ*E*_b_	Δ*E*_def_	Δ*E*_int_	Δ*E*_steric_ ^a^	Δ*E*_pol_ ^b^	Δ*E*_CT_	Δ*E*_CTπ//_	Δ*E*_CTσ**+**hy_ ^c^
B_2_(NHC)_2_-SiH_2_	–77.0	18.3	–95.3	337.6(−292.5)	–323.6(−238.3)	–109.3	–62.2	–47.1
B_2_(NHC)_2_-SiCl_2_	–63.8	18.7	–82.5	329.0(−290.3)	–276.8(−165.7)	–134.6	–76.0	–58.6
B_2_(Sil)_2_-SiH_2_	–81.6	8.5	–90.1	310.5(−283.7)	–282.4(−192.2)	–118.3	–65.8	–52.4
B_2_(Sil)_2_-SiCl_2_	–68.2	10.1	–78.3	307.2(−288.2)	–243.3(−129.1)	–142.3	–75.8	–66.5
CuCl-SiH_2_	–37.1	1.6	–38.7	–10.4(−55.9)	–10.3(−6.5)	–18.0	–3.2	–14.8(−13.4)
CuCl-SiCl_2_	–22.8	4.4	–27.2	0.1(−31.8)	–12.2(−9.0)	–15.0	–2.9	–12.1(−10.2)
AgCl-SiH_2_	–30.8	1.6	–32.4	3.0(−55.9)	–9.6(−5.0)	–25.8	–3.2	–22.6(−20.9)
AgCl-SiCl_2_	–17.0	4.3	–21.3	9.3(−31.6)	–9.9(−6.2)	–20.7	–2.9	–17.8(−15.7)
AuCl-SiH_2_	–59.2	3.1	–62.3	32.1(−109.4)	–21.7(−9.8)	–72.7	–9.2	–63.5(−58.2)
AuCl-SiCl_2_	–41.4	5.0	–46.3	38.7(−75.4)	–20.6(−10.1)	–64.5	–9.0	–55.4(−48.8)
CoCl(C_6_H_6_)-SiH_2_ ^d^	–43.2	7.8	–51.1	22.5(−88.6)	–20.7(−6.7)	–52.9	–13.3	–39.6

a Electrostatic interaction energies are given in parentheses.

b Values in parentheses are the polarization contributions originating from the SiR_2_ fragment.

c Coupling with π-backdonation is included. Values in parentheses correspond to the σ-donation components.

d Calculations were performed at the B3LYP-D3 level, as the M06-2X-D3 led to convergence difficulties.

Taking the deformed B_2_(NHC)_2_ and SiH_2_ as references, their interaction energy (Δ*E*_int_) reaches −95.3 kcal/mol. The term Δ*E*_steric_ (323.6 kcal/mol), which covers Pauli repulsion, electrostatic interactions, and electron correlation at the DFT level, is dominated by strong Pauli repulsion between the closed-shell valence orbitals of B_2_(NHC)_2_ and SiH_2_, since electrostatic interactions (−238.3 kcal/mol) contribute favorably to bonding. Such destabilizing Pauli repulsion induces subsequent electron-density relaxation within individual monomers in response to mutual electrostatic fields, corresponding to a prominent polarization stabilization (Δ*E*_pol_ = −323.6 kcal/mol). In addition, the very high charge-transfer energy (−109.3 kcal/mol) confirms the covalent nature of the B–Si linkage, similar to that of B–C in B_2_(NHC)_2_-CO. And this charge transfer increases the B–Si Wiberg bond index from 0.331 to 0.947.

Orbital interactions have traditionally been elucidated via MO correlation diagrams to visualize orbital energy shifts during complexation. The BLW method used here offers an additional layer of information. It enables the correlation of monomer orbitals even in the presence of other interacting fragments and tracks the orbital energy evolutions from isolated monomers, through their deformed and block-localized (diabatic) states in their mutual presence, to the final complex structure. Figure 4 shows the orbital interactions within the B–B–Si plane between B_2_(NHC)_2_ and SiH_2_. As reported previously, the ground state of SiH_2_ is a singlet (^1^A_1_) state, in which the central Si atom features a lone pair and an empty 3*p* orbital. And free B_2_(NHC)_2_ possesses degenerate HOMOs (Figure 4). Clearly, the HOMOs of B_2_(NHC)_2_ and SiH_2_ are symmetry-compatible, and the LUMO of SiH_2_ is also matched with the π_//_* orbital of B_2_(NHC)_2_. Nevertheless, effective chemical orbital interactions require electron donation from an occupied orbital of one fragment to a virtual orbital of the other, whereas pairwise occupied-occupied or unoccupied-unoccupied orbital cannot yield favorable orbital coupling. This paradox raises a critical and intriguing question regarding the bonding origin of the stabilized three-membered B–B–Si ring formed between B_2_(NHC)_2_ and SiH_2_.

**Figure 4:**
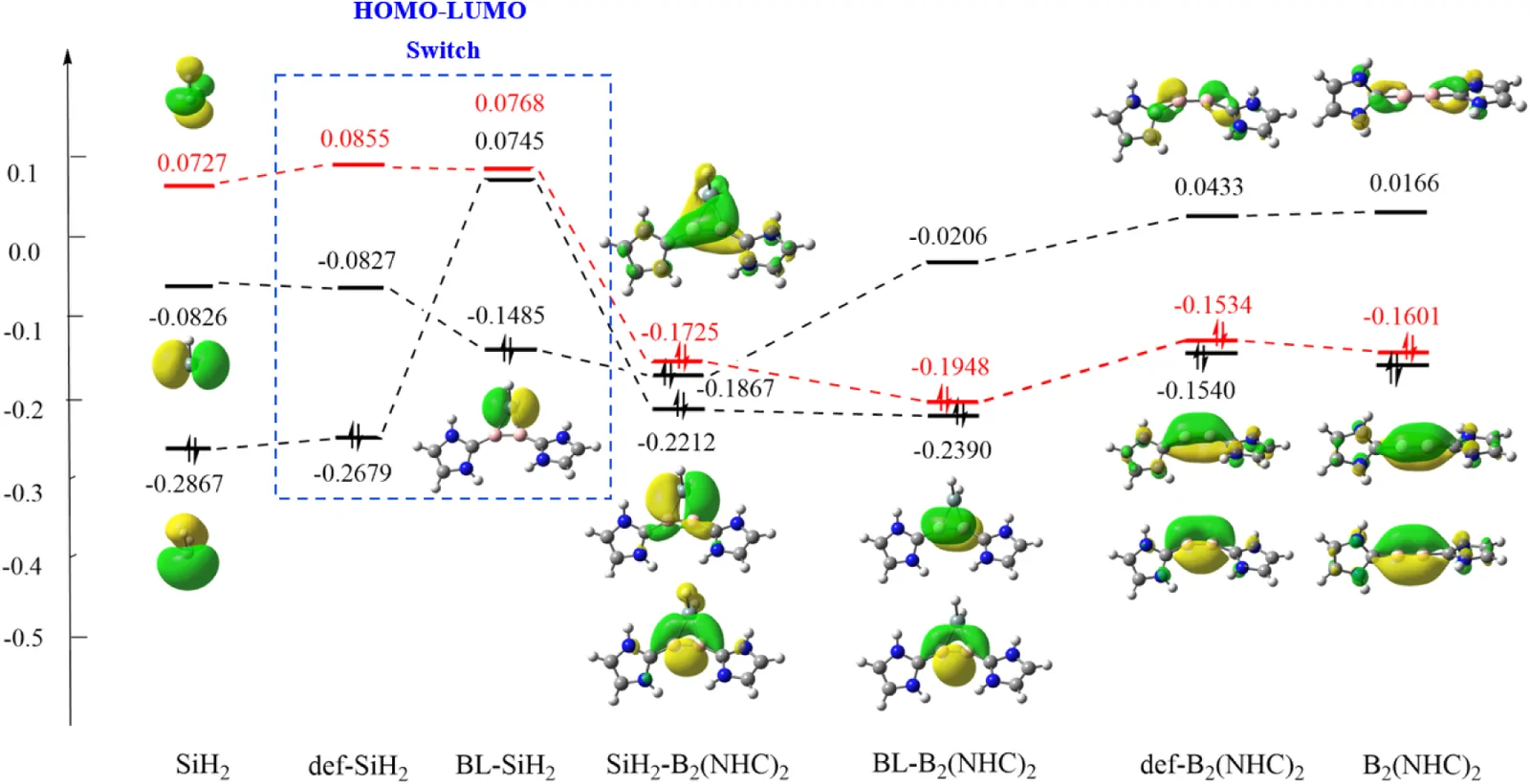
Complete orbital interaction diagrams for B_2_(NHC)_2_-SiH_2_, in which def- and BL- refer to deformed and block-localized, with the charge transfer quenched. The red lines are the π_⊥_ pathway, while the black lines represent both π_//_ and σ pathways. Energies are in atomic units.

As illustrated in Figure 4, geometric distortion of individual monomers toward their complex-bound conformations induces only minor variations in the HOMO and LUMO energies of B_2_(NHC)_2_ and SiH_2_. However, when these two deformed monomers are put together, there is not only a shift of energy levels, but also a reshuffling of the orders of energy levels. Notably, a HOMO–LUMO swap occurs for SiH_2_ fragment. It exactly corresponds to an electronic-structure feature arising from electron polarization triggered by steric (Pauli) repulsion from the approaching B_2_(NHC)_2_ whose HOMO is symmetrically compatible with the HOMO of SiH_2_. In other words, the SiH_2_ is in an excited electronic state. If we describe this excitation using isolated SiH_2_, it corresponds to the transition from the ground-state configuration [core]­(4a_1_)^2^(2b_2_)^2^(5a_1_)^2^ to the excited state [core]­(4a_1_)^2^(2b_2_)^2^(2b_1_)^2^, where [core] stands for (1a_1_)^2^(2a_1_)^2^(1b_1_)^2^(1b_2_)^2^(3a_1_).^2^ For the freely optimized SiH_2_, the excitation energy is calculated to be 71.7 kcal/mol at the M06-2X-D3/6-311+G­(d) level.

To visualize the polarization of B_2_(NHC)_2_ and SiH_2_, we plotted the electron density difference (EDD) maps (Figure 5a), in which orange and cyan regions represent electron density accumulation and depletion relative to the isolated monomers, respectively. Consistent with the above electronic structure analysis, electron density depletion is observed within the molecular plane of SiH_2_ (σ orbital direction), while electron density accumulation occurs in the direction perpendicular to SiH_2_ (π_//_ orbital direction). For B_2_(NHC)_2_, electron density variations are predominantly localized on the NHC ligands. Energetically, the polarization contribution originating from SiH_2_ reaches to −238.3 kcal mol^–1^, accounting for 73% of the total polarization energy.

**Figure 5:**
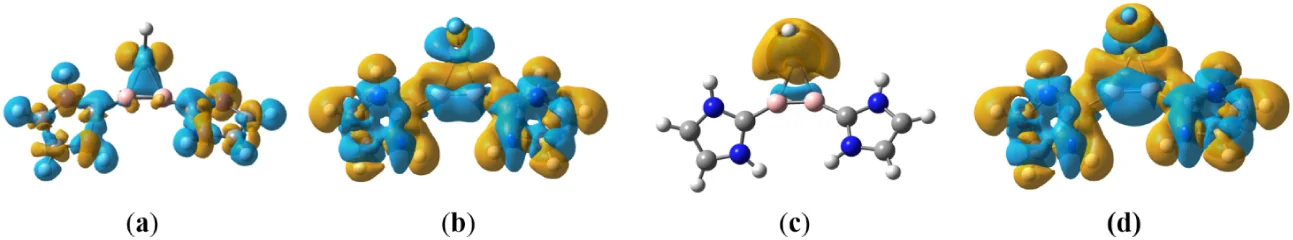
Electron density difference (EDD) maps showing the polarization (a) and electron transfer (b) from π_//_ orbital of SiH_2_ to B_2_(NHC)_2_, (c) from π_//_ and π_⊥_ orbitals of B_2_(NHC)_2_ to SiH_2_ and (d) between B_2_(NHC)_2_ and SiH_2_ in total. Isovalues are 0.02 a.u. for polarization and 0.005 a.u. for electron transfer, respectively.

Definitive hyperconjugation exists between the π orbital of B_2_(NHC)_2_ perpendicular to the B–B–Si ring (π_⊥_) and the SiH_2_ moiety. But orbital interactions within the B–B–Si plane require careful inspection. Following orbital rearrangement, these block-localized “*in situ*” frontier orbitals become ready for interaction. The HOMO of the diboryne overlaps effectively with the vacant σ orbital (originally HOMO but now LUMO) of SiH_2_, enabling efficient σ-donation. Meanwhile, the π lone-pair orbital (originally LUMO but now HOMO) of SiH_2_ overlaps with the π_//_* of the diboryne, completing the synergistic π-back-donation cycle. This orbital interaction pattern (Figure 4 and Scheme 1a) is fully consistent with the DCD bonding model widely used for transition-metal-ligand complexes. We note that in the B_2_(NHC)_2_-CO complex,^20^ the HOMO–LUMO swap occurs within the B_2_(NHC)_2_ fragment. But here, it occurs within SiH_2_, which displays distinct transition-metal-like behavior upon interaction with B_2_(NHC)_2_.

**Scheme 1:**
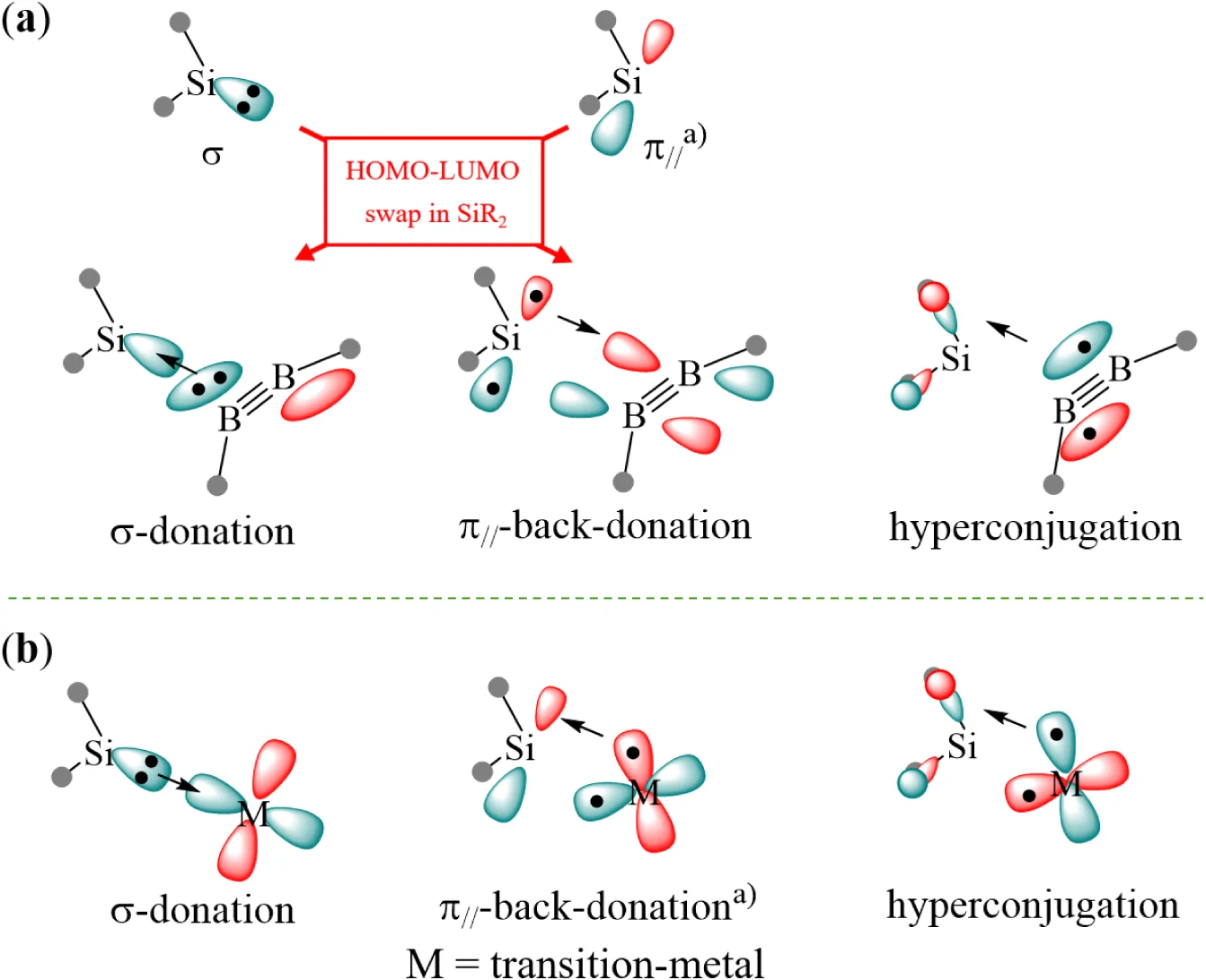
(a) Simplified Diagram Showing the Frontier Orbitals of Silylene and Its Bonding Mode with Diborynes, and (b) Schematic Bonding Modes in Transition-Metal Silylene Compounds^a^

a Although π_//_ is not strictly appropriate, it is retained for consistency with the B_2_L_2_-SiR_2_ system.

We further employed the BLW method to compute energy profiles against the Si···B distance for two distinct BLW (diabatic) states (Figure 6), where electrons of SiH_2_ and B_2_(NHC)_2_ are fully confined to their respective fragments. The BLW­(g-SiH_2_) state retains the ground-state electron configuration of isolated SiH_2_, while BLW­(e-SiH_2_) represents the excited SiH_2_ state. As shown in Figure 6, the two diabatic energy curves cross at approximately 2.9 Å. At equilibrium geometry of B_2_(NHC)_2_-SiH_2_, however, BLW­(e-SiH_2_) is energetically stabilized by 31.8 kcal/mol relative to BLW­(g-SiH_2_). This BLW­(e-SiH_2_) state is exactly the electronic state derived from BLW calculations at the equilibrium geometry discussed above. The BLW approach adopted herein is a single-determinant formalism, whereas multiconfigurational approaches are required for systems with strong multiconfigurational character. The sizable energy gap between BLW­(e-SiH_2_) and BLW­(g-SiH_2_) rules out notable multiconfigurational effects, firmly validating the reliability of our single-reference DFT methodology for the present system.

**Figure 6:**
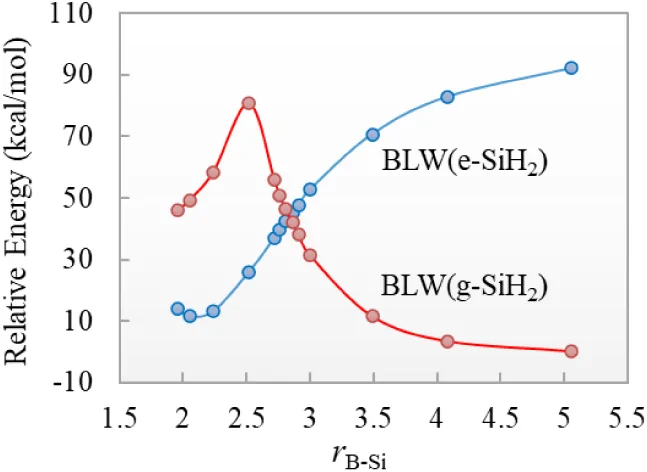
Energy profiles of two BLW (diabatic) states of B_2_(NHC)_2_-SiH_2_ versus Si···B distance.

Energetically, the π_//_-back-donation from SiH_2_ to B_2_(NHC)_2_ contributes a stabilization energy of −62.2 kcal/mol, accounting for more than half of the total charge-transfer energy (Δ*E*_CT_ = −109.3 kcal/mol). The combined σ-donation and hyperconjugation from B_2_(NHC)_2_ to SiH_2_ yields a stabilization energy of Δ*E*_CTσ+hy_ = −47.1 kcal/mol, which cannot be readily decomposed with delocalized NHC ligands. But the calculations based on localized NHCs constraints isolate the hyperconjugation contribution at −10.6 kcal/mol. Assuming negligible coupling between the σ-donation and hyperconjugation, the σ-donation energy is estimated to be −36.5 kcal/mol. In reality, the π bond of the B_2_ core can delocalize toward the NHCs, which weakens the hyperconjugation from the B_2_ core to SiH_2_. Consequently, delocalized NHCs reduce hyperconjugative stabilization below −10.6 kcal/mol and simultaneously raise σ-donation energy from B_2_(NHC)_2_ to SiH_2_ beyond −36.5 kcal/mol. Consistently, Figure 5b reveals significant electron flow from the π_//_ orbital of SiH_2_ to B_2_(NHC)_2_, which is accompanied by the excitation of SiH_2_ and electron-density perturbation over the NHC ligands. Figure 5c further illustrates the σ-donation and hyperconjugation from the π_//_ and π_⊥_ orbitals of B_2_(NHC)_2_ to SiH_2_.

Collectively, these energetic and orbital analyses confirm the hierarchy of three identified electron-transfer pathways (Scheme 1a). The π_//_-back-donation from SiH_2_ to B_2_(NHC)_2_ is dominant, followed by the σ-donation from B_2_(NHC)_2_ to SiH_2_, whereas hyperconjugation contributes the least. A similar conclusion holds for B_2_(Sil)_2_-SiH_2_ (see Table 1 and Figure S3), indicating that the ligand effect exerts negligible influence on the fundamental bonding model between diboryne and silylene.

To clarify the bonding differences between B_2_(NHC)_2_-SiH_2_ and transition-metal-SiH_2_ systems, we systematically analyzed a series of coinage-metal silylene MCl-SiH_2_ (M = Cu, Ag, Au). Structurally, the optimized M-Si bond lengths (2.285, 2.427, and 2.206 Å) show favorable consistency with experimental data.^26,30,61–65^ Meanwhile, in terms of bonding energetics, the trend AuCl > CuCl > AgCl for coinage-metal silylene complexes also coincides with Frenking’s theoretical results.^66^ The total binding energies of these MCl-SiH_2_ range from −30.8 to −59.2 kcal/mol. The steric term (Δ*E*_steric_) in these metal complexes is slightly positive or even negative (e.g., −10.4 kcal/mol in CuCl-SiH_2_), in sharp contrast to the large positive Pauli repulsion in diboryne systems. This behavior arises primarily from strong electrostatic interactions in these coinage-metal silylene complexes. Another notable difference lies in the charge-transfer contributions, especially π-back-donation. For CuCl-SiH_2_, AgCl-SiH_2_, and AuCl-SiH_2_ complexes, the π_//_-back-donation component Δ*E*_CTπ//_ is only −3.2 to −9.2 kcal/mol, while the hyperconjugation term ranges from −1.4 to −5.3 kcal/mol. Accordingly, charge transfer interactions in MCl-SiH_2_ complexes are overwhelmingly governed by σ-donation from the SiH_2_ to the metal center, which is distinctly different from typical transition-metal-CO adducts where π-backdonation prevails.^67,68^ These results demonstrate that SiH_2_ behaves as a classical σ-donor/π-acceptor ligand toward coinage-metals (Scheme 1b). Consistent with Frenking’s findings,^66^ the interaction is governed mainly by electrostatic attraction and σ-donation, with π-back-donation being nearly negligible.

In addition, we have also incorporated calculations of SiH_2_ coordinated to CoCl­(C_6_H_6_) to overcome limitations of closed-shell *d* ^10^ metal centers in the above MCl. Experimentally, stable analogue^69^ of CoCl­(C_6_H_6_)-silylene has been reported. The relevant energetic data have been summarized in Table 1. Although the π_//_-back-donation component Δ*E*_CTπ//_ within Δ*E*_CT_ is marked enhanced compared with that of MCl-SiH_2_ systems, electrostatic attraction still retains the dominant driving force for bonding. Therefore, our calculations reveal that varying transition-metal complexes only induces quantitative fluctuations in total interaction energies and individual energy components, rather than altering the intrinsic bonding nature.

In summary, consistent with the reported interaction between silylene and CO,^34,35^ SiH_2_ exhibits transition-metal-like character upon binding to diboryne, undergoing an initial excitation that enables stronger π-back-donation and more effective synergistic interactions. By contrast, SiH_2_ acts as a classical σ-donor/π-acceptor ligand toward transition-metals.

### Substituent Effect of Silylene

In the above discussions, SiH_2_ was adopted as a prototype silylene species to systematically explore the interactions between silylene and diboryne, or with transition-metals. Nevertheless, the experimental study reported by Xu et al.^23^ employed SiCl_2_ rather than SiH_2_. This raises a further mechanistic question regarding how substituents on the silylene modulate the bonding interactions with diboryne and transition-metal centers.

Chlorine substitution increases the positive charge on the Si atom in silylene, with the NPA charge rising from 0.554 to 0.869e. The enhanced positive charge on Si substantially weakens the overall electrostatic attraction between silylene and MCl. Specifically, the electrostatic interaction energies decrease by 24.1, 24.3, and 34.0 kcal/mol for CuCl, AgCl, and AuCl complexes, respectively (see Table 1). In terms of charge transfer, the increased positive charge on Si suppresses σ-donation capability toward MCl. Since the interaction between silylene and MCl is governed by electrostatic attraction and σ-electron transfer, the overall binding strength is consequently reduced.

However, the excitation energy of SiCl_2_ (144.7 kcal/mol) is significantly higher than that of SiH_2_ (71.7 kcal/mol). This is fully consistent with the reduction of the polarization term from −238.3 to −165.7 kcal/mol derived from energy decomposition analysis for SiR_2_. As a consequence, the total polarization contribution drops from −323.6 to −276.8 kcal/mol. On the other hand, the electron transfer from the lone pairs on Cl atoms to the 3*p* orbital of Si enhances the electron-donating ability of SiCl_2_ in its excited state through the π_//_ path. Meanwhile, Cl atoms also strengthen the electron-accepting capability of the empty orbitals in SiCl_2_, thereby promoting charge transfer from the π_//_ orbitals of diboryne to the empty orbitals of SiCl_2_. To gain insight into hyperconjugative interactions, we performed NBO analysis,^57–59^ which enables quantitative evaluation of orbital interaction energies via the second-order perturbation energy *E*(2). Although *E*(2) values cannot be directly compared with energy terms obtained from BLW-ED method, they are themselves directly comparable. The NBO analysis reveals orbital donation from the π_B–B_ orbital to σ*_Si–Cl_ antibonding orbital in B_2_(NHC)_2_-SiCl_2_ with an *E*(2) value of 48.0 kcal/mol. By comparison, the corresponding orbital donation from π_B–B_ to σ*_Si–H_ in B_2_(NHC)_2_-SiH_2_ yields a considerable *E*(2) value of 27.4 kcal/mol. Accordingly, the evident enhancement of hyperconjugative interactions further strengthens the overall charge transfer, which is confirmed by the charge-transfer energy from BLW-ED analysis increasing from −109.3 to −134.6 kcal/mol. Although both polarization and charge transfer are important in the silylene-diboryne system, the polarization effect, especially the polarization contribution of SiR_2_, is more significantly affected. As a result, the binding energy between SiR_2_ and diboryne is reduced from −77.0 to −63.8 kcal/mol. Therefore, due to their fundamentally distinct bonding natures, chlorine substitution imposes a less pronounced impact on silylene–diboryne interactions compared with silylene-MCl complexes.

## Conclusions

In summary, we systematically investigated the bonding nature and substituent effects of the complexes B_2_L_2_-SiR_2_ (L = NHC, silylene; R = H, Cl) using DFT and BLW-ED methods. Energy decomposition analyses reveal that the diboryne-SiH_2_ interaction is governed by considerable electrostatic attraction, polarization, and charge transfer. A spontaneous HOMO–LUMO inversion occurs within SiH_2_ upon approaching B_2_(NHC)_2_, exciting SiH_2_ to a higher electronic state and enabling synergistic DCD-type interactions. In this scenario, SiH_2_ behaves as a transition-metal-like center, with π_//_-back-donation serving as the dominant charge-transfer contribution. In contrast, transition-metal silylene complexes show distinctly different bonding characters. The interactions therein are governed primarily by electrostatic attraction, and SiH_2_ behaves as a classical σ-donor/π-acceptor ligand with negligible π-back-donations. Notably, chlorine substitution has opposite effects on the two types of interactions: it significantly weakens the metal-silylene interaction by suppressing electrostatic attraction and σ-donation, whereas it only moderately reduces the diboryne-silylene interaction by lowering polarization despite enhanced charge transfer. This work thus unveils the versatile bonding behavior of silylenes, which can act as either transition-metal-like centers toward diborynes or classical ligands toward transition-metals. Such versatile bonding characteristics render silylenes a highly active area of research in contemporary inorganic chemistry, opening new directions in main-group chemistry. Our results will deepen the understanding of main-group metallomimetic chemistry and offer valuable theoretical guidance for the rational design of novel low-valent main-group complexes.

## Supplementary Material



## Notes

The influences of substituents, basis sets, and density functionals on optimized geometries, interaction energies, and bonding nature; the initial model, constrained-optimized and free-optimized simple models of B_2_(Sil)_2_-SiR_2_; NBOs of B_2_(NHC)_2_ and B_2_(Sil)_2_; complete orbital interaction diagrams for B_2_(Sil)_2_-SiH_2_; energy profiles of two BLW states of B_2_(Sil)_2_-SiH_2_ versus Si···B distance; optimized structures of MCl-SiR_2_ (M = Cu, Ag, Au; R = H, Cl) and CoCl­(C_6_H_6_)-SiH_2_; Cartesian coordinates of studied compounds (PDF)
