# Supplementary material for: Bonding Nature of B2L2‑SiR2 (L = NHC, Silylene; R = H, Cl): Metallomimetic, Silylene, or Diboryne?
Source: Inorg Chem. 2026 Aug 12;65(33):19247–55. doi: 10.1021/acs.inorgchem.6c02220 (PMC13508999; doi:10.1021/acs.inorgchem.6c02220)
Supplement: Supplementary file 1 [file ic6c02220_si_001.pdf]

Supporting Information

Bonding Nature of B_2L_2-SiR_2 (L = NHC, Silylene; R = H, Cl): Metallomimetic, Silylene or Diboryne?

Huaiyu Zhang,[*,a] Rui Ma,[a] Huixuan Sun,[a] Jinshuai Song,[b] Yandong Duan,[c] Yirong Mo[*,d]

[a] Institute of Computational Quantum Chemistry, and Hebei Key Laboratory of Inorganic Nanomaterials, College of Chemistry and Materials Science, Hebei Normal University, Shijiazhuang, 050024, China

[b] Green Catalysis Center, and College of Chemistry, Zhengzhou University, Zhengzhou, 450001, China

[c] Hebei Key Laboratory of Photoelectric Control on Surface and Interface, School of Sciences, Hebei University of Science and Technology, Shijiazhuang, 050018, China

[d] Department of Nanoscience, Joint School of Nanoscience and Nanoengineering, University of North Carolina at Greensboro, Greensboro, North Carolina, 27401, United States

The corresponding author emails: huaiyu.zhang@hebtu.edu.cn, y_mo3@uncg.edu.

1. The influences of substituents, basis sets, and density functionals on optimized geometries， interaction energies and bonding nature.
2. The initial model, final (constrained optimized) and free optimized simple model of $B_2(Sil)_2$-SiR_2.
3. NBOs of $B_2(NHC)_2$ and $B_2(Sil)_2$.
4. Complete orbital interaction diagrams for $B_2(Sil)_2$-SiH_2.
5. Energy profiles of two BLW states of $B_2(Sil)_2$-SiH_2 versus Si···B distance.
6. Optimized structures of MCl-SiR_2 (M=Cu, Ag, Au; R=H, Cl) and CoCl(C_6H_6)-SiH_2.
7. Cartesian coordinates of studied compounds at different level.

1. The influences of substituents, basis sets, and density functionals on optimized geometries，interaction energies and bonding nature.

Table S1. Key geometrical parameters in the optimized compounds with different substituents, basis sets and density functions.

	Basis set	DFT	r_{B-B}	r_{B-L_C}[a]	$\angle L_CBB$[a]			
$B_2(IDep)_2$ [b]	*exp*	*exp*	1.446(3)	1.484(3)<br>1.488(3)	175.4(2)<br>172.0(2)			
$B_2(IDep)_2$[b]	Def2-TZVP	M06-2X-D3	1.447	1.480<br>1.479	167.6<br>170.5			
$B_2(NHC)_2$	Def2-TZVP	M06-2X-D3	1.444	1.487<br>1.487	180.0<br>180.0			
$B_2(NHC)_2$	6-311+G(d)	M06-2X-D3	1.444	1.488<br>1.488	180.0<br>180.0			
$B_2(NHC)_2$	6-311+G(d)	B3LYP-D3	1.452	1.478<br>1.478	180.0<br>180.0			
	Basis set	DFT	r_{B-B}	r_{B-L_C}[a]	$\angle L_CBB$[a]	r_{B-Si}	$\angle SiBL_C$	ΔE[c]
$B_2(IDep)_2$-SiH_2	Def2-TZVP	M06-2X-D3	1.530	1.509<br>1.516	165.5<br>150.7	1.986<br>1.923	139.3<br>127.0	-80.9
$B_2(NHC)_2$-SiH_2	Def2-TZVP	M06-2X-D3	1.540	1.519<br>1.519	151.7<br>151.7	1.951<br>1.951	141.0<br>141.0	-79.7
$B_2(NHC)_2$-SiH_2	6-311+G(d)	M06-2X-D3	1.537	1.521<br>1.521	153.5<br>153.5	1.962<br>1.962	139.3<br>139.3	-78.6
$B_2(NHC)_2$-SiH_2	6-311+G(d)	B3LYP-D3	1.548	1.510<br>1.510	154.4<br>154.4	1.976<br>1.976	138.6<br>138.6	-69.3
$B_2(IDep)_2$-$SiCl_2$	Def2-TZVP	M06-2X-D3	1.558	1.521<br>1.524	155.8<br>156.4	1.896<br>1.887	133.6<br>136.2	-71.9
$B_2(NHC)_2$- $SiCl_2$	Def2-TZVP	M06-2X-D3	1.571	1.526<br>1.526	147.7<br>147.6	1.888<br>1.888	146.3<br>146.3	-65.4
$B_2(NHC)_2$- $SiCl_2$	6-311+G(d)	M06-2X-D3	1.571	1.528<br>1.528	148.9<br>148.9	1.893<br>1.893	145.0<br>145.0	-67.8
$B_2(NHC)_2$- $SiCl_2$	6-311+G(d)	B3LYP-D3	1.583	1.517<br>1.517	149.2<br>149.2	1.904<br>1.904	144.5<br>144.5	-56.7
	Basis set	DFT	r_{B-B}	$r_{B-L_{Si}}$[a]	$\angle L_{Si}BB$[a]			
$B_2(Sil^R)_2$ [b]	*exp*	*exp*	1.444(4)<br>-<br>1.459(3)	1.869(2)<br>-<br>1.885(2)	164.4(2)-<br>177.39(2)			
$B_2(Sil^R)_2$ [b]	Def2-TZVP	M06-2X-D3						

$B_2(Sil)_2$ initial model	Def2-TZVP	M06-2X-D3	1.449	1.869<br>1.869	159.3<br>159.3			
$B_2(Sil)_2$ initial model	6-311+G(d)	M06-2X-D3	1.449	1.870<br>1.870	159.0<br>159.0			
$B_2(Sil)_2$ initial model	6-311+G(d)	B3LYP-D3	1,449	1.870<br>1.870	158.8<br>158.8			
$B_2(Sil)_2$ final model	6-311+G(d)	M06-2X-D3	1.449	1.870<br>1.870	159.0<br>159.0			
	Basis set	DFT	$r_{B\text{-}B}$	$r_{B\text{-}L_{Si}}$[a]	∠$L_{Si}BB$[a]	$r_{B\text{-}Si}$	∠$SiBL_{Si}$	$⊿E$[c]
$B_2(Sil^R)_2$- SiH_2	Def2-TZVP	M06-2X-D3	1.535	1.877<br>1.870	159.0<br>164.5	1.951<br>1.945	128.7<br>133.7	-80.1
$B_2(Sil)_2$-SiH_2 initial model	Def2-TZVP	M06-2X-D3	1.528	1.869<br>1.869	167.5<br>167.6	1.959<br>1.958	125.2<br>125.3	-86.1
$B_2(Sil)_2$-SiH_2 initial model	6-311+G(d)	M06-2X-D3	1.527	1.871<br>1.871	166.7<br>166.7	1.965<br>1.966	125.7<br>125.8	-76.0
$B_2(Sil)_2$-SiH_2 initial model	6-311+G(d)	B3LYP-D3	1.535	1.871<br>1.871	162.7<br>162.7	1.967<br>1.967	130.0<br>130.1	-74.7
$B_2(Sil)_2$-SiH_2 final model	6-311+G(d)	M06-2X-D3	1.527	1.871<br>1.871	166.7<br>166.7	1.965<br>1.966	125.7<br>125.8	-83.4
$B_2(Sil^R)_2$- $SiCl_2$ [b]	exp	exp	1.580(3)			1.888(2)<br>1.895(2)		
$B_2(Sil^R)_2$- $SiCl_2$ [b]	Def2-TZVP	M06-2X-D3	1.569	1.884<br>1.893	160.9<br>157.3	1.889<br>1.891	133.5<br>136.9	-71.9
$B_2(Sil)_2$- $SiCl_2$ initial model	Def2-TZVP	M06-2X-D3	1.562	1.884<br>1.884	164.1<br>164.1	1.898<br>1.898	130.2<br>130.2	-74.4
$B_2(Sil)_2$- $SiCl_2$ initial model	6-311+G(d)	M06-2X-D3	1.561	1.887<br>1.887	164.3<br>164.3	1.902<br>1.902	129.8<br>129.8	-69.2
$B_2(Sil)_2$- $SiCl_2$ initial model	6-311+G(d)	B3LYP-D3	1.565	1.883<br>1.883	162.4<br>162.4	1.903<br>1.903	131.8<br>131.8	-67.2
$B_2(Sil)_2$- $SiCl_2$ final model	6-311+G(d)	M06-2X-D3	1.561	1.887<br>1.887	164.3<br>164.3	1.902<br>1.902	129.8<br>129.8	-72.4

a. L_C/L_{Si} is the carbon/silicon atom linking to the B_2 core on diboryne ligands.

b. Experimentally reported molecules.

c. BSSE corrections are not included. The $⊿E$s are gotten using Gaussian 16 at different levels.

To validate the reliability of our truncated models and computational strategy, we systematically evaluated the influences of substituent truncation, basis set selection, and density functional choice on the optimized geometric parameters and interaction energies.

Two representative basis sets (6-311+G(d) and def2-TZVP) and two widely applied functionals (M06-2X-D3 and B3LYP-D3) were tested for comparative verification, and all computational results obtained at different theoretical levels are compiled in Table S1. Comprehensive calculations demonstrate that variations in substituents, density functionals, and basis sets only induce only minor quantitative discrepancies in structural parameters. Specifically, the B-B and B-Si bond lengths fluctuate by less than 0.1 Å, and Si-B-$L_{C/Si}$ bond angles deviate within 10 degrees.

Table S2. Computed energy components (kcal/mol) with the BLW-ED approach at M06-2X-D3/6-311+G(d) level.[a]

	$r_{B\text{-}Si}$	$\angle SiBL_{C/Si}$	ΔE_b	ΔE_{def}	ΔE_{int}	ΔE_{steric}	ΔE_{pol}	ΔE_{CT}	$\Delta E_{CT\pi//}$	$\Delta E_{CT\sigma+hy}$	$\Delta E'^{b}$	$\Delta E_{CT\pi//}/\Delta E_{CT\sigma+hy}$
$B_2(NHC)_2$-SiH_2	1.870	139.0	-74.0	18.3	-92.3	408.7	-388.4	-112.6	-67.7	-44.9	-28.3	1.5
	1.916		-76.2	18.3	-94.5	371.6	-355.0	-111.1	-65.1	-46.1	-28.4	1.4
	**1.962**		**-77.0**	**18.3**	**-95.3**	**337.6**	**-323.6**	**-109.3**	**-62.2**	**-47.0**	**-31.8**	**1.3**
	2.008		-76.5	18.3	-94.8	306.6	-294.3	-107.1	-59.2	-47.9	-34.9	1.2
	2.054		-75.0	18.3	-93.3	278.2	-266.9	-104.6	-56.1	-48.6	-37.7	1.2
	1.962	125	-61.4	17.5	-78.9	321.7	-300.3	-100.2	-52.6	-47.6	-54.9	1.1
		130	-64.2	18.7	-83.0	343.7	-324.6	-102.0	-55.4	-46.6	-48.9	1.2
		135	-66.2	20.6	-86.8	402.3	-385.1	-104.0	-58.4	-45.6	-42.8	1.3
		**139**	**-77.0**	**18.3**	**-95.3**	**337.6**	**-323.6**	**-109.3**	**-62.2**	**-47.0**	**-31.8**	**1.3**
$B_2(Sil)_2$-SiH_2	1.874	125.8	-78.5	8.5	-87.0	376.2	-340.6	-122.5	-72.8	-49.7	-18.1	1.5
	1.919		-80.8	8.5	-89.3	341.9	-310.6	-120.6	-69.4	-51.1	-21.7	1.4
	**1.965**		**-81.6**	**8.5**	**-90.1**	**310.5**	**-282.4**	**-118.3**	**-65.8**	**-52.4**	**-25.0**	**1.3**
	2.011		-81.2	8.5	-89.7	281.9	-256.0	-115.6	-62.1	-53.5	-28.0	1.2
	2.058		-79.8	8.5	-88.3	255.7	-231.3	-112.7	-58.4	-54.4	-30.5	1.1
	1.965	**125.8**	**-81.6**	**8.5**	**-90.1**	**310.5**	**-282.4**	**-118.3**	**-65.8**	**-52.4**	**-25.0**	**1.3**
		130.0	-79.2	14.5	-93.7	306.8	-279.5	-121.0	-69.4	-51.6	-18.2	1.3
		135.0	-78.1	15.6	-93.7	321.0	-277.2	-137.5	-87.4	-50.1	-16.9	1.7
		140.0	-76.7	16.8	-93.6	302.2	-275.4	-120.4	-71.7	-48.7	-15.7	1.5

a. Values shown in bold were obtained at equilibrium geometries.

b. $\Delta E' = E_{BLW(e\text{-}SiH2)} - E_{BLW(g\text{-}SiH2)}$

Additional BLW-ED calculations (see Table S2) with manually perturbed key geometric parameters were then performed to further verify that minor structural deviations in B-B bond lengths and Si-B-$L_{C/Si}$ angles originating from substituent truncation do not alter the core bonding conclusions of this work. Key structural parameters were manually perturbed around the equilibrium geometries. The B-Si bond was elongated and shortened by approximately 0.05 and 0.1 Å, respectively. The ∠Si-B-$L_{C/Si}$ bond angles were gradually reduced (for $B_2(NHC)_2$-SiH_2) or increased (for $B_2(Sil)_2$-SiH_2) by 15 degrees to match the corresponding values extracted from complete experimental structures. Our computations demonstrate that the characteristic HOMO-LUMO inversion phenomenon persists. At different B–Si distances and ∠Si-B-$L_{C/Si}$ bond angles near the equilibrium geometry, the ratio $\Delta E_{CT\pi//}/\Delta E_{CT\sigma+hy}$ remains above 1.0, albeit declining with increasing B-Si bond length and decreasing ∠Si-B-$L_{C/Si}$ bond angle. It means $\pi_{//}$-backdonation is dominant remains invariant within the geometric uncertainty introduced by substituent truncation. Accordingly, the truncated molecular geometries optimized at the M06-2X-D3/6-311+G(d) level are physically reasonable, and minor structural perturbations do not modify the fundamental bonding nature of the studied complexes.

Table S3. Computed energy components (kcal/mol) with the BLW-ED approach at different levels.

		ΔE_b	ΔE_{def}	ΔE_{int}	ΔE_{steric}	ΔE_{pol}	ΔE_{CT}	$\Delta E_{CT\pi//}$	$\Delta E_{CT\sigma+hy}$	$\Delta E_{CT\pi//}/\Delta E_{CT\sigma+hy}$
6-311+G(d)	M06-2X-D3	-77.0	18.3	-95.3	337.6	-323.6 (-238.3)	-109.3	-62.2	-47.1	1.3
6-311+G(d)	B3LYP-D3	-65.7	18.9	-84.7	338.7	-312.0 (-223.1))	-111.3	-63.6	-47.7	1.3
6-311+G(d)	PBE0-D3	-77.8	18.9	-96.7	368.7	-326.8 (-234.3)	-138.6	-84.9	-53.7	1.6
6-311+G(d)	wB97X-D	-80.4	18.2	-98.6	334.4	-322.2 (-239.0)	-110.8	-63.4	-47.4	1.3
Def2-TZVP	M06-2X-D3	-78.9	18.1	-97.0	338.8	-367.9 (-263.1)	-67.9	-44.3	-23.6	1.9

a. Values in parentheses are the polarization contributions originating from the SiH_2 fragment.

Table 3 takes $B_2(NHC)_2$-SiH_2 as a representative example to examine the influences of density functionals and basis sets on energy decomposition results. Although the B3LYP-D3 functional underestimates the overall interaction energies and the def2-TZVP basis set yields slightly reduced absolute values of ΔE_{CT}, these comparative calculations unambiguously verify the robustness of the key bonding features. Specifically, the characteristic HOMO-LUMO inversion remains persistent, the dominant polarization contribution originating from the SiH_2 fragment is consistently preserved, and the qualitative proportions of σ-donation and π-backdonation components are well maintained across different theoretical levels.

Therefore, truncated substituent models are reasonably adopted throughout this work. Unless otherwise noted, all structural, energetic, and bonding discussions in the main text are based on computational data obtained at the M06-2X-D3/6-311+G(d) theoretical level.

2. The initial model, final (constrained optimized) and free optimized simple model of $B_2(Sil)_2$-SiR_2.

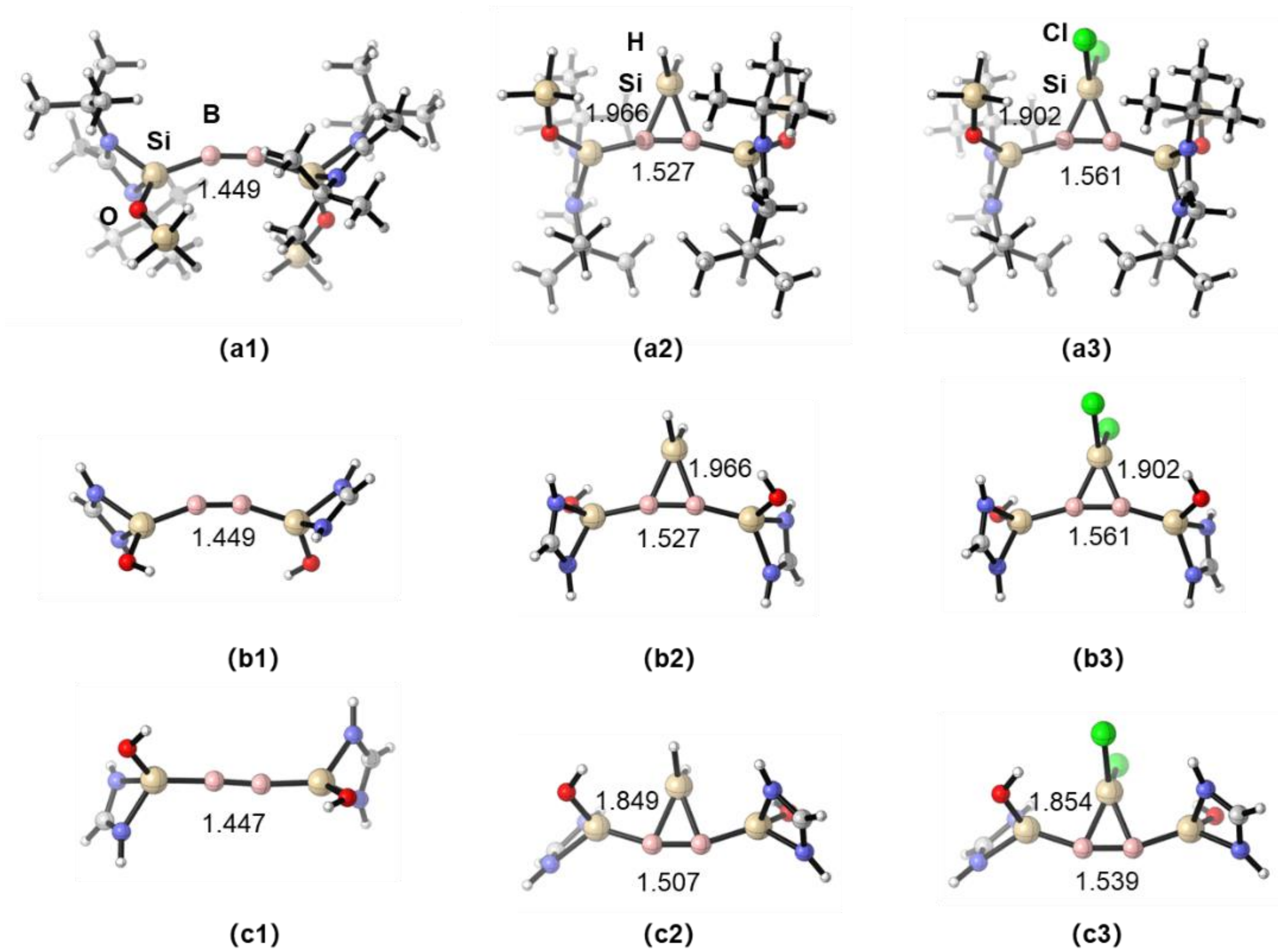

Figure S1. The structures of **(a)** initial model, **(b)** constrained optimized and **(c)** free optimized simple model of $B_2(Sil)_2$-SiR_2 computed at the M06-2X-D3/6-311+G(d) level.

The steric bulk of substituents on the silylene exerts only a slight influence on the B-B and B-Si bond lengths in both the diboryne and $B_2(Sil)_2$-SiR_2 compounds, but significantly affects the ∠SiBSi angles. To reduce computational cost while retaining key electronic characteristics, the constrained simplified model was employed to analyze the diboryne-silylene (B_2L_2-SiR_2) interaction.

3. NBOs of $B_2(NHC)_2$ and $B_2(Sil)_2$.

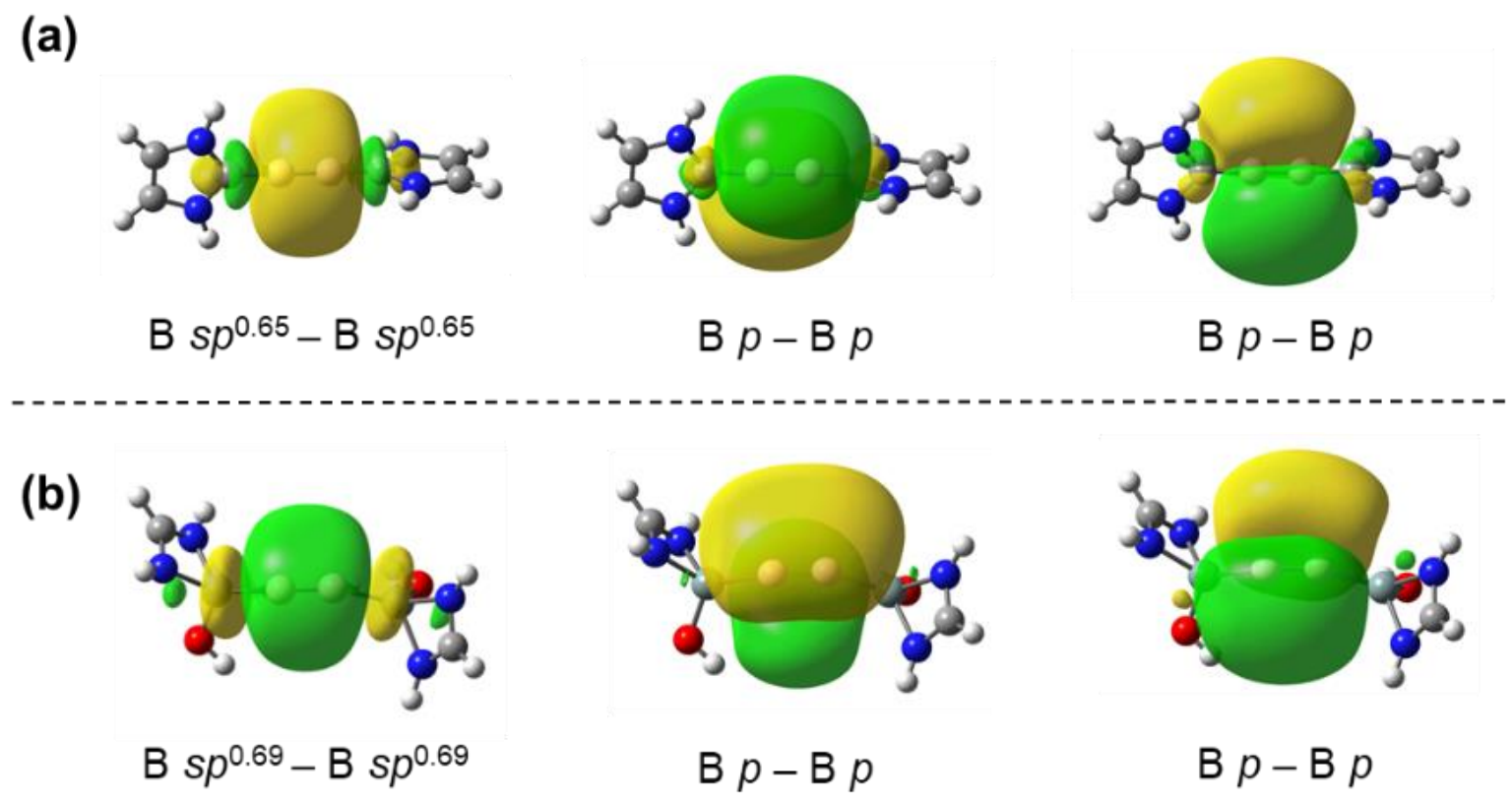

Figure S2. NBOs of (a) $B_2(NHC)_2$ and (b) $B_2(Sil)_2$.

4. Complete orbital interaction diagrams for $B_2(Sil)_2$-SiH_2.

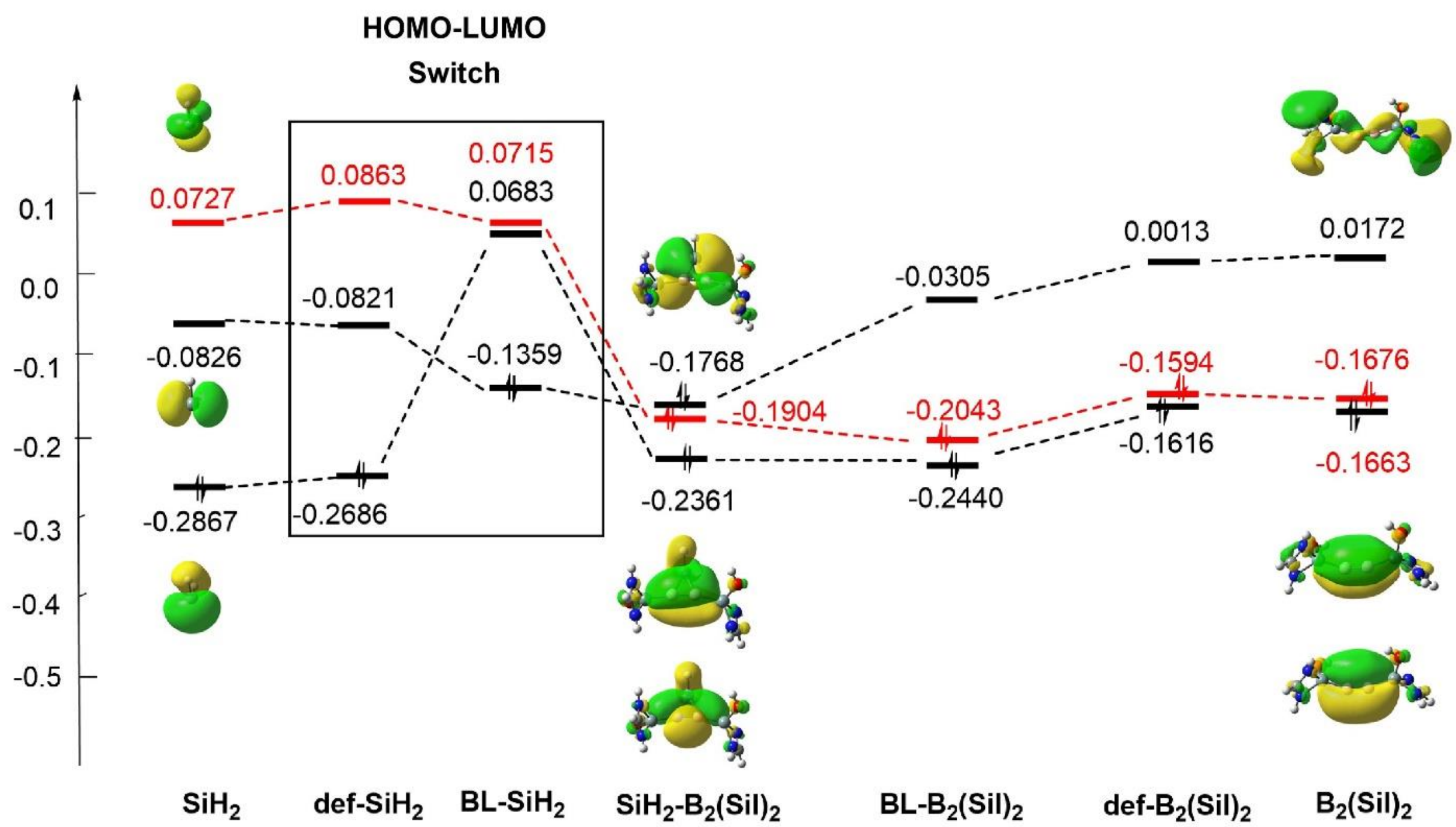

Figure S3. Complete orbital interaction diagrams for $B_2(Sil)_2$-SiH_2, in which def- and BL- refer to deformed and block-localized with the charge transfer quenched. The red lines are the $\pi_{\perp}$ pathway, while the black lines represent both $\pi_{//}$ and σ pathways. Energies are at the atomic units.

5. Energy profiles of two BLW states of $B_2(SiI)_2$-SiH_2 versus Si···B distance.

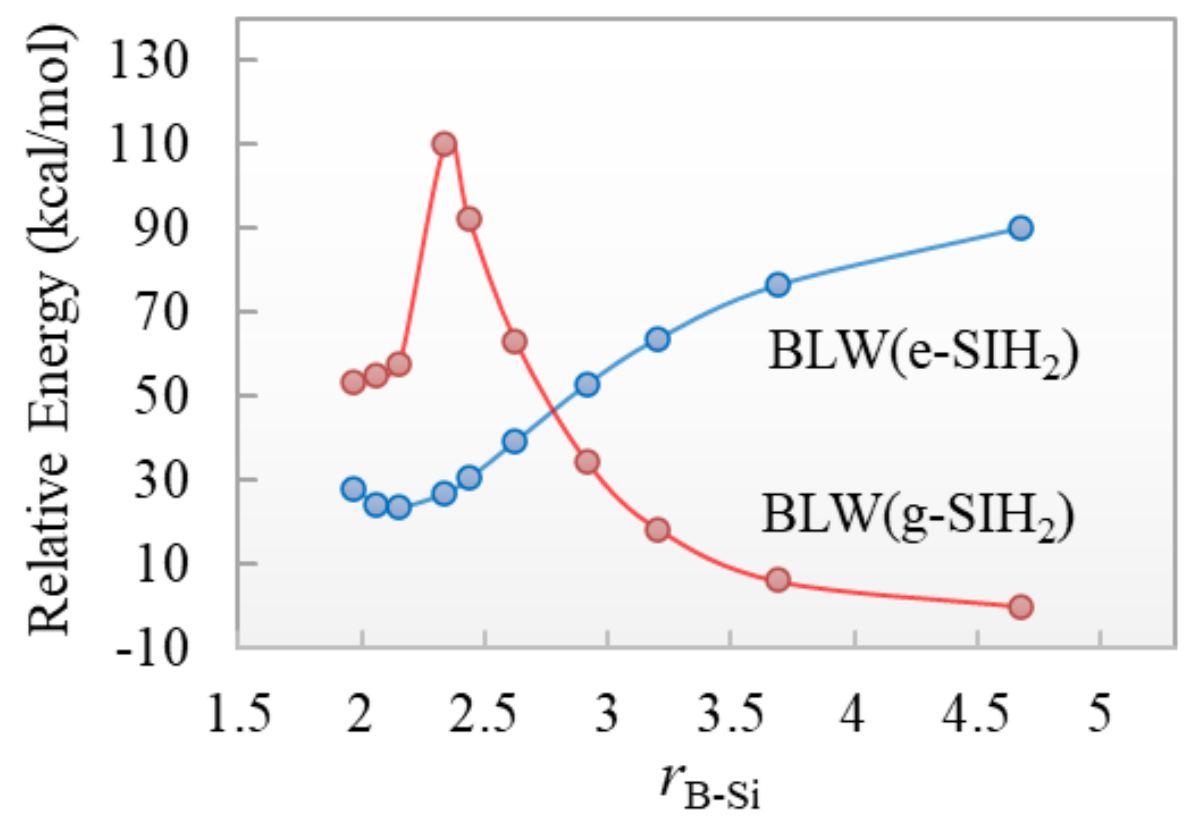

Figure S4. Energy profiles of two BLW states of $B_2(SiI)_2$-SiH_2 versus Si···B distance.

6. Optimized structures of MCl-SiR$_2$ (M=Cu, Ag, Au; R=H, Cl) and $CoCl(C_6H_6)$-SiH_2

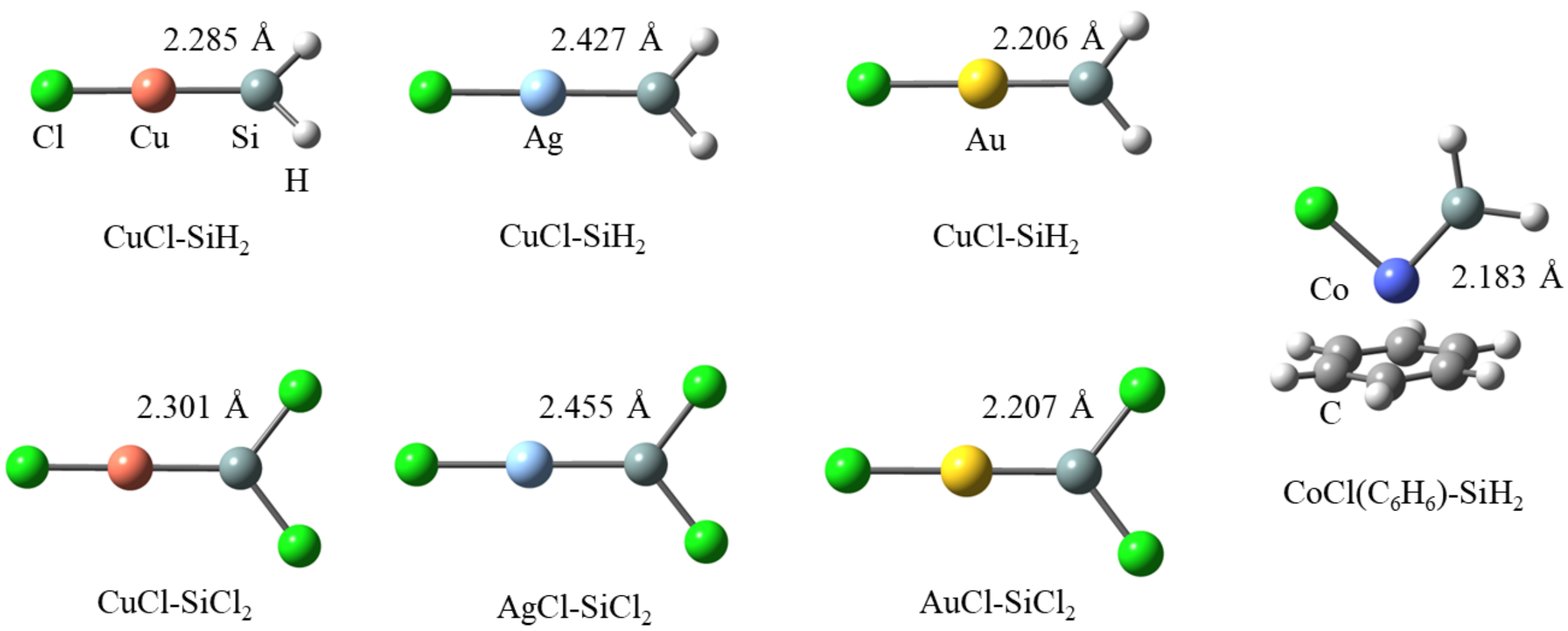

Figure S5. Optimized structures of MCl-SiR$_2$ (M=Cu, Ag, Au; R=H, Cl) and $CoCl(C_6H_6)$-SiH_2 of with M-Si bond lengths (Å) computed at the M06-2X-D3/6-311+G(d) level.

7. Cartesian coordinates of studied compounds at different level.

(1) SiH_2 M06-2X-D3/6-311+G(d)

Si	14.0	0.000000	0.761405	-0.000000
H	1.0	1.085539	-0.297203	0.000000
H	1.0	-1.085539	-0.297203	-0.000000

(2) $SiCl_2$ M06-2X-D3/6-311+G(d)

Si	14.0	0.000000	0.943261	-0.000000
CL	17.0	1.609091	-0.388131	0.000000
CL	17.0	-1.609091	-0.388131	-0.000000

(3) the full experimental structure $B_2(IDep)_2$ M06-2X-D3/Def2-TZVP

B	5.0	-0.719593	0.407036	-0.233810
B	5.0	0.558813	-0.181896	-0.568771
C	6.0	-4.156443	1.316620	0.835434
C	6.0	-3.433451	1.733881	1.892114
H	1.0	-5.216200	1.329068	0.655514
H	1.0	-3.733552	2.198213	2.814586
C	6.0	-1.986143	0.856619	0.386718
N	7.0	-3.269063	0.779068	-0.083826
N	7.0	-2.107282	1.437701	1.625112
C	6.0	3.586995	-2.304302	-0.533117
C	6.0	4.102428	-1.171274	-1.042797
H	1.0	4.046266	-3.249436	-0.303617
H	1.0	5.103040	-0.926003	-1.351150
N	7.0	2.237585	-2.079565	-0.308818
N	7.0	3.074826	-0.243372	-1.110974
C	6.0	1.900310	-0.796395	-0.669253
C	6.0	3.211029	1.098312	-1.580604
C	6.0	3.192604	2.153494	-0.666314
C	6.0	3.334583	1.291252	-2.960080
C	6.0	3.354839	3.439294	-1.177154
C	6.0	3.499516	2.591473	-3.422084
C	6.0	3.518107	3.657118	-2.534197
H	1.0	3.344837	4.277936	-0.491447
H	1.0	3.595100	2.779242	-4.482692
H	1.0	3.643520	4.665568	-2.907473
C	6.0	1.367170	-2.946402	0.422000
C	6.0	1.468591	-2.947455	1.817219
C	6.0	0.432850	-3.716762	-0.266560

C	6.0	0.643928	-3.816070	2.523979
C	6.0	-0.382442	-4.562654	0.480715
C	6.0	-0.270488	-4.621405	1.859898
H	1.0	0.702213	-3.855020	3.603736
H	1.0	-1.113962	-5.175877	-0.031578
H	1.0	-0.908145	-5.289425	2.425294
C	6.0	-3.621045	0.133156	-1.307891
C	6.0	-3.799636	0.905882	-2.451274
C	6.0	-3.738709	-1.262005	-1.307313
C	6.0	-4.163242	0.251292	-3.626138
C	6.0	-4.103884	-1.873647	-2.499789
C	6.0	-4.322435	-1.122061	-3.647570
H	1.0	-4.304275	0.831750	-4.530412
H	1.0	-4.214645	-2.948650	-2.540352
H	1.0	-4.602640	-1.618516	-4.567939
C	6.0	-0.985305	1.762217	2.448077
C	6.0	-0.402257	0.764007	3.226077
C	6.0	-0.476502	3.061571	2.376908
C	6.0	0.701546	1.114480	4.000345
C	6.0	0.618029	3.372771	3.175899
C	6.0	1.198696	2.407199	3.985920
H	1.0	1.176366	0.355403	4.611543
H	1.0	1.038202	4.369134	3.150129
H	1.0	2.056738	2.662360	4.595358
C	6.0	3.231695	0.101005	-3.886514
H	1.0	4.077769	-0.569887	-3.710764
C	6.0	2.977468	1.947866	0.812222
H	1.0	2.047184	1.389779	0.962906
C	6.0	-3.606787	2.399141	-2.437619
H	1.0	-2.867955	2.666703	-1.680381
C	6.0	-3.425268	-2.038493	-0.051067
H	1.0	-3.932652	-1.565210	0.795101
C	6.0	0.302148	-3.638615	-1.764161
H	1.0	0.327026	-2.589980	-2.069561
C	6.0	2.393799	-1.966023	2.497944
H	1.0	3.366333	-1.978922	2.001037
C	6.0	-0.917473	-0.650628	3.216697
H	1.0	-1.038435	-0.977682	2.179859
C	6.0	-1.087682	4.038959	1.400989
H	1.0	-2.132729	4.215607	1.673163
C	6.0	-4.912200	3.157362	-2.191643

H	1.0	-4.749020	4.234807	-2.230835
H	1.0	-5.658087	2.895848	-2.943813
H	1.0	-5.325489	2.915542	-1.211924
C	6.0	4.128883	1.247110	1.533714
H	1.0	5.064728	1.795590	1.412058
H	1.0	3.908258	1.178780	2.601115
H	1.0	4.280111	0.232952	1.162465
C	6.0	3.158454	0.438292	-5.367918
H	1.0	3.037616	-0.472855	-5.953081
H	1.0	2.310474	1.090301	-5.581781
H	1.0	2.310474	1.090301	-5.581781
H	1.0	4.065398	0.936691	-5.713440
C	6.0	2.605238	-2.183874	3.988933
H	1.0	3.306335	-1.443695	4.375568
H	1.0	1.679484	-2.088123	4.558233
H	1.0	3.015922	-3.175123	4.189602
C	6.0	1.382550	-4.435323	-2.496839
H	1.0	1.363901	-5.484416	-2.197048
H	1.0	1.232127	-4.385318	-3.575738
H	1.0	2.375002	-4.038475	-2.279114
C	6.0	-3.777881	-3.516976	-0.096893
H	1.0	-3.209045	-4.036908	-0.869486
H	1.0	-3.535863	-3.985652	0.857103
H	1.0	-4.839778	-3.673746	-0.295836
C	6.0	-2.219106	-0.842486	3.995208
H	1.0	-2.504834	-1.895521	3.996877
H	1.0	-2.107618	-0.516975	5.030901
H	1.0	-3.037053	-0.277285	3.547751
C	6.0	-0.359640	5.368088	1.276288
H	1.0	0.675957	5.218643	0.965913
H	1.0	-0.845233	5.992519	0.527119
H	1.0	-0.356700	5.918661	2.218333
H	1.0	2.819432	2.928593	1.264831
H	1.0	-1.113951	3.544438	0.424319
H	1.0	2.343200	-0.467573	-3.594670
H	1.0	-3.191767	2.698751	-3.401509
H	1.0	-2.351611	-1.921660	0.143904
H	1.0	-0.682995	-4.020031	-2.040772
H	1.0	-0.147569	-1.291209	3.648499
H	1.0	1.990071	-0.959887	2.314983

(4) $B_2(NHC)_2$ M06-2X-D3/Def2-TZVP

B	5.0	0.722191	0.000053	0.000043
B	5.0	-0.722191	0.000040	0.000005
C	6.0	4.383443	-0.476024	-0.475685
H	1.0	2.718623	-1.425437	-1.424545
C	6.0	4.383404	0.476242	0.475967
H	1.0	2.718505	1.425611	1.424733
H	1.0	5.200026	-0.969780	-0.969118
H	1.0	5.199944	0.970021	0.969445
C	6.0	2.209416	0.000074	0.000085
N	7.0	3.060179	-0.757315	-0.756819
N	7.0	3.060115	0.757497	0.757027
C	6.0	-4.383415	0.475866	-0.476196
H	1.0	-2.718538	1.424714	-1.425522
C	6.0	-4.383432	-0.475852	0.476004
H	1.0	-2.718590	-1.424661	1.425429
H	1.0	-5.199967	0.969343	-0.969957
H	1.0	-5.200003	-0.969345	0.969718
N	7.0	-3.060133	0.756963	-0.757445
N	7.0	-3.060161	-0.756923	0.757327
C	6.0	-2.209416	0.000026	-0.000037

(5) $B_2(NHC)_2$ M06-2X-D3/6-311+G(d)

B 5.0	0.721820	0.000331	-0.000596
B 5.0	-0.721822	0.000178	-0.000501
C 6.0	4.389663	0.477600	-0.476060
H 1.0	2.722590	1.430388	-1.425855
C 6.0	4.389272	-0.478200	0.476652
H 1.0	2.721419	-1.430388	1.425672
H 1.0	5.206421	0.973805	-0.970606
H 1.0	5.205625	-0.974685	0.971586
C 6.0	2.209927	0.000072	-0.000233
N 7.0	3.063537	0.759988	-0.757595
N 7.0	3.062915	-0.760121	0.757560
C 6.0	-4.389377	0.476352	0.478289
H 1.0	-2.721730	1.425723	1.430489
C 6.0	-4.389561	-0.476200	-0.477672
H 1.0	-2.722283	-1.425497	-1.430592
H 1.0	-5.205837	0.971000	0.974882
H 1.0	-5.206212	-0.970879	-0.973920
N 7.0	-3.063081	0.757628	0.760132

N	7.0	-3.063374	-0.757410	-0.760095
C	6.0	-2.209929	0.000124	-0.000163

(6) $B_2(NHC)_2$ B3LYP-D3/6-311+G(d)

B	5.0	0.726218	0.000064	0.000035
B	5.0	-0.726218	0.000049	0.000024
C	6.0	4.396775	-0.479734	-0.477008
H	1.0	2.734400	-1.438275	-1.429988
C	6.0	4.396795	0.479694	0.476975
H	1.0	2.734461	1.438267	1.429999
H	1.0	5.215058	-0.976259	-0.970678
H	1.0	5.215106	0.976185	0.970635
C	6.0	2.204483	0.000016	-0.000005
N	7.0	3.068463	-0.765629	-0.761266
N	7.0	3.068502	0.765614	0.761272
C	6.0	-4.396781	0.476973	-0.479736
H	1.0	-2.734418	1.430123	-1.438132
C	6.0	-4.396789	-0.476990	0.479712
H	1.0	-2.734443	-1.430113	1.438163
H	1.0	-5.215076	0.970643	-0.976241
H	1.0	-5.215087	-0.970684	0.976188
N	7.0	-3.068479	0.761270	-0.765615
N	7.0	-3.068487	-0.761288	0.765609
C	6.0	-2.204482	0.000005	0.000021

(7) $B_2(IDep)_2$-SiH_2 M06-2X-D3/Def2-TZVP

B	5.0	0.567009	0.020182	0.701437
B	5.0	-0.825071	0.531919	0.323753
C	6.0	3.348964	-2.429217	1.129964
C	6.0	3.973627	-1.274724	1.423366
H	1.0	3.692657	-3.448060	1.150294
H	1.0	4.976425	-1.074658	1.756132
C	6.0	1.855614	-0.755557	0.820272
N	7.0	2.055907	-2.106507	0.741911
N	7.0	3.064329	-0.255657	1.210165
C	6.0	-2.921328	1.571067	-2.590771
C	6.0	-3.869078	1.360341	-1.655646
H	1.0	-2.990960	1.896855	-3.613205
H	1.0	-4.938858	1.459373	-1.694524
N	7.0	-1.708020	1.241827	-2.014267
N	7.0	-3.220086	0.925931	-0.517173

C	6.0	-1.875482	0.861132	-0.718645
C	6.0	-3.867993	0.531401	0.697268
C	6.0	-4.187094	1.506925	1.635327
C	6.0	-4.115995	-0.829518	0.895142
C	6.0	-4.836828	1.095919	2.796225
C	6.0	-4.766556	-1.195367	2.067047
C	6.0	-5.131659	-0.238906	3.005496
H	1.0	-5.088317	1.834986	3.547743
H	1.0	-4.986068	-2.236815	2.258105
H	1.0	-5.631847	-0.544755	3.915470
C	6.0	-0.457864	1.298994	-2.707496
C	6.0	0.138673	2.549861	-2.876329
C	6.0	0.101518	0.117585	-3.187900
C	6.0	1.300919	2.611351	-3.637008
C	6.0	1.277630	0.224053	-3.928317
C	6.0	1.858533	1.458059	-4.169760
H	1.0	1.790058	3.562777	-3.796458
H	1.0	1.737161	-0.678579	-4.315308
H	1.0	2.766399	1.521717	-4.756678
C	6.0	1.147644	-3.038231	0.147753
C	6.0	0.061760	-3.509279	0.881734
C	6.0	1.399021	-3.428057	-1.172168
C	6.0	-0.777796	-4.433201	0.263649
C	6.0	0.544254	-4.363203	-1.744556
C	6.0	-0.536302	-4.862226	-1.030501
H	1.0	-1.628109	-4.817637	0.813451
H	1.0	0.708026	-4.691731	-2.762324
H	1.0	-1.197118	-5.585749	-1.491508
C	6.0	3.300405	1.127908	1.476084
C	6.0	3.452808	2.008541	0.406171
C	6.0	3.316763	1.537971	2.811034
C	6.0	3.694617	3.344751	0.710743
C	6.0	3.559698	2.882907	3.067107
C	6.0	3.758631	3.775906	2.025538
H	1.0	3.820456	4.052979	-0.099373
H	1.0	3.569694	3.242426	4.086752
H	1.0	3.942809	4.820582	2.241618
C	6.0	-3.632521	-1.836147	-0.121779
H	1.0	-3.917940	-1.497065	-1.122057
C	6.0	-3.826309	2.953967	1.432545
H	1.0	-2.927625	3.022430	0.816947

C	6.0	-0.186140	-3.068643	2.298425
H	1.0	-0.025453	-1.993280	2.384983
C	6.0	2.519457	-2.767596	-1.942987
H	1.0	3.449878	-2.849206	-1.376786
C	6.0	-0.499036	-1.232449	-2.893455
H	1.0	-0.623604	-1.333632	-1.809566
C	6.0	-0.460020	3.752873	-2.186066
H	1.0	-1.452733	3.953946	-2.600216
C	6.0	3.318210	1.563924	-1.028184
H	1.0	2.349134	1.070273	-1.160273
C	6.0	3.025285	0.537798	3.905663
H	1.0	3.897585	-0.108345	4.051237
C	6.0	0.698343	-3.812443	3.300758
H	1.0	0.475409	-3.490327	4.318180
H	1.0	0.542382	-4.890796	3.238386
H	1.0	1.754096	-3.611601	3.110449
C	6.0	-4.964476	3.766660	0.813407
H	1.0	-5.863127	3.705224	1.429135
H	1.0	-4.684895	4.816476	0.719817
H	1.0	-5.215402	3.395836	-0.181188
C	6.0	-4.126738	-3.258672	0.089476
H	1.0	-3.744489	-3.905987	-0.700121
H	1.0	-3.780999	-3.662714	1.042173
H	1.0	-5.216970	-3.310842	0.077364
C	6.0	0.384798	5.015795	-2.247675
H	1.0	-0.093044	5.813556	-1.680379
H	1.0	1.372822	4.844953	-1.816634
H	1.0	0.515471	5.367322	-3.272260
C	6.0	-1.818131	-1.532558	-3.605671
H	1.0	-1.716309	-1.415770	-4.686130
H	1.0	-2.118909	-2.561433	-3.400121
H	1.0	-2.622628	-0.880142	-3.268222
C	6.0	2.756503	-3.299080	-3.348583
H	1.0	1.881255	-3.180094	-3.989612
H	1.0	3.582801	-2.761156	-3.813860
H	1.0	3.013174	-4.359686	-3.333013
C	6.0	4.439200	0.652092	-1.527460
H	1.0	4.290107	0.426940	-2.585547
H	1.0	5.413901	1.129562	-1.413249
H	1.0	4.460881	-0.295264	-0.987896
C	6.0	2.613311	1.152553	5.235445

H	1.0	1.753537	1.810805	5.099678
H	1.0	2.330775	0.368099	5.936968
H	1.0	3.420997	1.727557	5.690286
H	1.0	-3.564903	3.376296	2.404434
H	1.0	2.220142	-0.112890	3.552571
H	1.0	-2.534699	-1.822078	-0.106062
H	1.0	-1.238177	-3.242323	2.534579
H	1.0	2.302907	-1.691061	-1.980378
H	1.0	0.229199	-1.985916	-3.190503
H	1.0	3.287030	2.459113	-1.650968
H	1.0	-0.625004	3.475103	-1.139606
Si	14.0	-0.438812	0.898622	2.171582
H	1.0	-0.870037	0.081935	3.349333
H	1.0	-0.093255	2.250536	2.707303

(8) $B_2(NHC)_2$-SiH_2 M06-2X-D3/Def2-TZVP

Si	14.0	-0.000008	2.408148	0.000025
H	1.0	0.141743	3.299927	1.188263
H	1.0	-0.141738	3.299939	-1.188206
B	5.0	0.768838	0.615633	-0.040787
B	5.0	-0.768844	0.615634	0.040827
C	6.0	4.319248	-0.528745	-0.244029
H	1.0	3.354230	1.275409	-0.893309
C	6.0	3.773227	-1.582320	0.400150
H	1.0	1.733950	-1.870388	0.984322
H	1.0	5.333361	-0.343972	-0.546816
H	1.0	4.222279	-2.485823	0.769565
C	6.0	2.110724	-0.095140	0.000692
N	7.0	3.294243	0.362034	-0.471549
N	7.0	2.429579	-1.301015	0.533513
C	6.0	-3.773192	-1.582381	-0.400094
H	1.0	-1.733888	-1.870466	-0.984163
C	6.0	-4.319269	-0.528723	0.243904
H	1.0	-3.354282	1.275464	0.893144
H	1.0	-4.222214	-2.485928	-0.769438
H	1.0	-5.333406	-0.343916	0.546589
N	7.0	-2.429545	-1.301063	-0.533437
N	7.0	-3.294272	0.362058	0.471452
C	6.0	-2.110725	-0.095148	-0.000684

(9) $B_2(NHC)_2$-SiH_2 M06-2X-D3/6-311+G(d)

Si	14.0	0.00000000	0.00000000	0.00000000
H	1.0	1.19980623	0.89363700	0.00000000
H	1.0	-1.19980623	0.89363800	0.00000000
B	5.0	0.04158050	-1.80479047	-0.76752642
B	5.0	-0.04157004	-1.80480653	0.76747370
C	6.0	0.24142755	-2.79902705	-4.36693726
H	1.0	-0.34457373	-0.96668028	-3.40315131
C	6.0	0.68043302	-3.94298042	-3.79124597
H	1.0	0.93066559	-4.37296929	-1.70719245
H	1.0	0.11073588	-2.54107023	-5.40344809
H	1.0	1.00965547	-4.86875810	-4.23038121
C	6.0	0.20788597	-2.47756620	-2.12107733
N	7.0	-0.03461387	-1.92532884	-3.33623560
N	7.0	0.64695408	-3.72805984	-2.42657891
C	6.0	-0.68037990	-3.94315558	3.79109014
H	1.0	-0.93049519	-4.37309171	1.70701200
C	6.0	-0.24166936	-2.79912095	4.36684673
H	1.0	0.34430556	-0.96672373	3.40313883
H	1.0	-1.00948679	-4.86899789	4.23017861
H	1.0	-0.11113438	-2.54115678	5.40337546
N	7.0	-0.64689473	-3.72817316	2.42643475
N	7.0	0.03443787	-1.92539916	3.33618316
C	6.0	-0.20791648	-2.47763280	2.12099461

(10) $B_2(NHC)_2$-SiH_2 B3LYP-D3/6-311+G(d)

B	5.0	-0.773629	0.524372	-0.021940
B	5.0	0.773627	0.524380	0.021659
C	6.0	-4.403086	-0.363646	-0.082136
H	1.0	-3.361648	1.509200	-0.299013
C	6.0	-3.878349	-1.596784	0.136726
H	1.0	-1.822057	-2.157717	0.339289
H	1.0	-5.427324	-0.046146	-0.182012
H	1.0	-4.360018	-2.551656	0.265949
C	6.0	-2.136114	-0.126821	-0.005619
N	7.0	-3.335661	0.509170	-0.163157
N	7.0	-2.503340	-1.435674	0.176440
C	6.0	3.878383	-1.596745	-0.136676
H	1.0	1.822140	-2.157628	-0.339879
C	6.0	4.403071	-0.363646	0.082527
H	1.0	3.361587	1.509156	0.299550
H	1.0	4.360081	-2.551592	-0.265972

H	1.0	5.427286	-0.046165	0.182692
N	7.0	2.503386	-1.435620	-0.176719
N	7.0	3.335627	0.509155	0.163479
C	6.0	2.136117	-0.126804	0.005526
Si	14.0	-0.000017	2.342516	-0.000100
H	1.0	-0.071680	3.235500	1.203449
H	1.0	0.071668	3.235524	-1.203627

(11) $B_2(IDep)_2$-$SiCl_2$ M06-2X-D3/Def2-TZVP

B	5.0	0.634890	-0.117234	-0.482972
B	5.0	-0.733993	-0.391403	0.209288
C	6.0	2.860139	2.209611	-2.385305
C	6.0	3.710278	1.207691	-2.084364
H	1.0	2.991659	3.105044	-2.966230
H	1.0	4.740964	1.047132	-2.345532
C	6.0	1.735114	0.725381	-1.116927
N	7.0	1.653803	1.910812	-1.775215
N	7.0	3.015625	0.314099	-1.297113
C	6.0	-2.831525	-0.069782	3.262115
C	6.0	-3.749110	-0.509426	2.375817
H	1.0	-2.900268	0.134277	4.315518
H	1.0	-4.785164	-0.770503	2.495819
N	7.0	-1.662108	0.143986	2.554434
N	7.0	-3.121019	-0.576405	1.147647
C	6.0	-1.823466	-0.200666	1.253499
C	6.0	-3.701213	-1.036332	-0.078682
C	6.0	-3.680504	-2.398603	-0.356611
C	6.0	-4.204001	-0.079127	-0.964565
C	6.0	-4.241411	-2.813832	-1.562465
C	6.0	-4.763612	-0.538993	-2.149084
C	6.0	-4.785627	-1.897359	-2.441939
H	1.0	-4.220027	-3.867272	-1.814499
H	1.0	-5.177805	0.163691	-2.859018
H	1.0	-5.213152	-2.236119	-3.376869
C	6.0	-0.432884	0.620567	3.111638
C	6.0	0.374857	-0.289867	3.794396
C	6.0	-0.078143	1.951252	2.903042
C	6.0	1.537683	0.202177	4.378544
C	6.0	1.101811	2.396855	3.494207
C	6.0	1.888635	1.537298	4.244312
H	1.0	2.190988	-0.469901	4.918283

H	1.0	1.404347	3.428509	3.352119
H	1.0	2.799839	1.901831	4.702219
C	6.0	0.543577	2.814731	-1.714564
C	6.0	-0.574837	2.587510	-2.513159
C	6.0	0.649358	3.898415	-0.836140
C	6.0	-1.605164	3.522560	-2.444358
C	6.0	-0.399060	4.810869	-0.809823
C	6.0	-1.516712	4.625059	-1.611268
H	1.0	-2.484715	3.376741	-3.058804
H	1.0	-0.352922	5.665078	-0.147516
H	1.0	-2.326811	5.342863	-1.578972
C	6.0	3.543938	-0.917404	-0.786887
C	6.0	3.861829	-1.009068	0.566158
C	6.0	3.655435	-1.988144	-1.676340
C	6.0	4.398124	-2.214663	1.010061
C	6.0	4.190600	-3.173104	-1.185465
C	6.0	4.575459	-3.278308	0.142533
H	1.0	4.654903	-2.319886	2.057225
H	1.0	4.280247	-4.031593	-1.836173
H	1.0	4.981906	-4.211406	0.510659
C	6.0	-4.071609	1.389025	-0.632965
H	1.0	-4.397998	1.554580	0.397663
C	6.0	-3.077490	-3.406129	0.584876
H	1.0	-2.351844	-2.922958	1.240199
C	6.0	-0.674689	1.398506	-3.431144
H	1.0	-0.338865	0.500538	-2.909394
C	6.0	1.837208	3.995603	0.094383
H	1.0	2.759786	3.883896	-0.478971
C	6.0	-0.885636	2.863379	2.015176
H	1.0	-0.995053	2.389141	1.033312
C	6.0	0.002592	-1.753847	3.811304
H	1.0	-0.849813	-1.907598	4.481300
C	6.0	3.580495	0.106008	1.542494
H	1.0	2.518326	0.368592	1.486167
C	6.0	3.128844	-1.846900	-3.085909
H	1.0	3.755834	-1.142429	-3.642232
C	6.0	0.115268	1.586636	-4.727370
H	1.0	-0.010493	0.720359	-5.376955
H	1.0	-0.220781	2.473721	-5.266817
H	1.0	1.181502	1.698194	-4.523904
C	6.0	-4.138994	-4.127343	1.416821

H	1.0	-4.863093	-4.630096	0.774044
H	1.0	-3.679633	-4.873920	2.064874
H	1.0	-4.686021	-3.422310	2.045773
C	6.0	-4.825858	2.331605	-1.557496
H	1.0	-4.668822	3.364848	-1.246849
H	1.0	-4.481916	2.240892	-2.588534
H	1.0	-5.898285	2.129729	-1.544111
C	6.0	1.137985	-2.694431	4.185364
H	1.0	0.814431	-3.727938	4.069031
H	1.0	1.990707	-2.541685	3.520573
H	1.0	1.466308	-2.557371	5.216867
C	6.0	-2.257724	3.269108	2.553092
H	1.0	-2.172315	3.716899	3.544564
H	1.0	-2.712104	4.002861	1.885156
H	1.0	-2.937140	2.420304	2.620944
C	6.0	1.931449	5.272562	0.916322
H	1.0	1.077560	5.403505	1.582601
H	1.0	2.829815	5.249424	1.533357
H	1.0	1.987857	6.152745	0.273604
C	6.0	4.435588	1.360910	1.366737
H	1.0	4.200610	2.082906	2.151609
H	1.0	5.498837	1.123099	1.428005
H	1.0	4.252683	1.846669	0.407744
C	6.0	3.010457	-3.151554	-3.858677
H	1.0	2.373056	-3.856934	-3.323777
H	1.0	2.557409	-2.966163	-4.832080
H	1.0	3.983671	-3.615927	-4.025618
H	1.0	-2.512550	-4.128488	-0.008873
H	1.0	2.138068	-1.384364	-3.019542
H	1.0	-3.003002	1.639836	-0.647403
H	1.0	-1.729169	1.236265	-3.664019
H	1.0	1.807253	3.121825	0.759991
H	1.0	-0.297408	3.765924	1.852008
H	1.0	3.735305	-0.289317	2.547725
H	1.0	-0.344719	-2.017805	2.807823
Si	14.0	0.074245	-1.916878	-0.573544
Cl	17.0	-0.550003	-2.941684	-2.323551
Cl	17.0	0.842078	-3.544111	0.554573

(12) $B_2(NHC)_2$-$SiCl_2$ M06-2X-D3/Def2-TZVP

Si	14.0	0.000015	1.520313	0.000104

Cl	17.0	-0.165327	2.866664	-1.617704
Cl	17.0	0.167253	2.866312	1.617985
B	5.0	-0.784076	-0.196226	0.056610
B	5.0	0.783327	-0.196801	-0.056583
C	6.0	-4.213548	-1.654001	0.378781
H	1.0	-3.307271	0.061679	1.286289
C	6.0	-3.674986	-2.515039	-0.510006
H	1.0	-1.696088	-2.515079	-1.327830
H	1.0	-5.198688	-1.612840	0.805286
H	1.0	-4.102328	-3.363868	-1.010967
C	6.0	-2.079353	-1.002720	0.020780
N	7.0	-3.226722	-0.742298	0.684323
N	7.0	-2.372952	-2.103082	-0.707596
C	6.0	3.675202	-2.513773	0.512258
H	1.0	1.698235	-2.510183	1.334754
C	6.0	4.211780	-1.656467	-0.381301
H	1.0	3.303742	0.055691	-1.293602
H	1.0	4.103607	-3.360584	1.015717
H	1.0	5.195926	-1.617170	-0.810269
N	7.0	2.373644	-2.100872	0.711166
N	7.0	3.224341	-0.745886	-0.688308
C	6.0	2.078484	-1.003422	-0.020984

(13) $B_2(NHC)_2$-$SiCl_2$ M06-2X-D3/6-311+G(d)

Si	14.0	0.00000000	0.00000000	0.00000000
Cl	17.0	1.63814284	1.3550985	0.0000000
Cl	17.0	-1.63814284	1.3550557	0.0000000
B	5.0	0.02303352	-1.72227771	-0.78469330
B	5.0	-0.02303635	-1.72193518	0.78573564
C	6.0	0.08944840	-3.07366223	-4.27658623
H	1.0	-0.83574774	-1.32822039	-3.43822844
C	6.0	0.85023959	-4.00278541	-3.65313763
H	1.0	1.39872751	-4.10254383	-1.58291261
H	1.0	-0.19866978	-2.97868993	-5.30895793
H	1.0	1.35837359	-4.86967958	-4.03805384
C	6.0	0.19190916	-2.50154392	-2.08805369
N	7.0	-0.29661391	-2.17118945	-3.30610742
N	7.0	0.89228158	-3.63802660	-2.32002735
C	6.0	-0.85181569	-3.99875377	3.65660337
H	1.0	-1.40351311	-4.09819580	1.58720580
C	6.0	-0.08766740	-3.07133269	4.27844917

H	1.0	0.84080830	-1.32870534	3.43792184
H	1.0	-1.36170220	-4.86408712	4.04271339
H	1.0	0.20219101	-2.97657828	5.31035374
N	7.0	-0.89477547	-3.63462252	2.32333665
N	7.0	0.29941159	-2.17039380	3.30692979
C	6.0	-0.19175679	-2.50009784	2.08974744

(14) $B_2(NHC)_2$-$SiCl_2$ B3LYP-D3/6-311+G(d)

B	5.0	0.788204	-0.247604	0.073242
B	5.0	-0.787884	-0.247821	-0.072738
C	6.0	4.304247	-1.510192	0.305007
H	1.0	3.387358	0.302973	0.998202
C	6.0	3.737043	-2.537206	-0.376370
H	1.0	1.702523	-2.756117	-1.012900
H	1.0	5.320642	-1.356847	0.625910
H	1.0	4.166986	-3.443526	-0.768195
C	6.0	2.098996	-1.009775	0.044611
N	7.0	3.296117	-0.595337	0.548686
N	7.0	2.395122	-2.219523	-0.516736
C	6.0	-3.737143	-2.536784	0.377189
H	1.0	-1.703396	-2.754398	1.016652
C	6.0	-4.303556	-1.511065	-0.306779
H	1.0	-3.385985	0.300938	-1.002057
H	1.0	-4.167522	-3.442393	0.770174
H	1.0	-5.319561	-1.358368	-0.629223
N	7.0	-2.395398	-2.218777	0.518607
N	7.0	-3.295178	-0.596587	-0.550896
C	6.0	-2.098651	-1.010028	-0.044570
Si	14.0	0.000035	1.484333	0.000193
Cl	17.0	0.216309	2.832539	-1.654626
Cl	17.0	-0.217097	2.832523	1.654865

(15) the full experimental structure $B_2(Sil^R)_2$ M06-2X-D3/ Def2-TZVP

Si	14.0	2.413628	-0.474503	0.431838
Si	14.0	-2.590256	0.220975	0.271210
Si	14.0	4.055372	2.316378	0.500703
Si	14.0	-4.599997	-2.273632	0.555649
O	8.0	3.423574	0.798588	0.502979
O	8.0	-3.818243	-0.841864	0.299858
N	7.0	3.463094	-1.915999	0.914101
N	7.0	-2.835026	1.249189	-1.236073

N	7.0	-3.248222	1.872305	0.776255
N	7.0	2.863105	-1.433252	-1.091991
C	6.0	-3.142607	2.297339	-0.484247
C	6.0	3.483930	-2.345464	-0.337450
C	6.0	4.538165	2.694257	2.318586
C	6.0	-3.191714	2.650046	2.030454
C	6.0	4.069567	-2.453787	2.139326
C	6.0	-2.560769	1.131018	-2.675524
C	6.0	-3.296082	3.696365	-0.954593
C	6.0	2.201520	-1.628255	-2.399842
C	6.0	5.577669	2.154351	-0.664746
C	6.0	4.099326	-3.602413	-0.831667
C	6.0	2.729797	3.539873	-0.150725
C	6.0	1.681976	3.829058	0.935092
H	1.0	1.246271	2.905644	1.327287
H	1.0	0.867322	4.414300	0.490951
H	1.0	2.081235	4.417096	1.760466
C	6.0	-1.839160	3.355398	2.161697
H	1.0	-1.034166	2.632873	2.008032
H	1.0	-1.744239	3.785116	3.160884
H	1.0	-1.739552	4.165748	1.440104
C	6.0	3.375208	2.325365	3.261560
H	1.0	2.600164	3.087667	3.276046
H	1.0	3.754983	2.229844	4.284224
H	1.0	2.891054	1.383864	2.995240
C	6.0	6.301481	0.822542	-0.397458
H	1.0	6.771525	0.779566	0.582945
H	1.0	7.091753	0.689131	-1.144807
H	1.0	5.611025	-0.021160	-0.481090
C	6.0	3.571205	-3.870658	2.430740
H	1.0	2.480742	-3.898308	2.393833
H	1.0	3.891497	-4.165627	3.431298
H	1.0	3.973020	-4.595240	1.724639
C	6.0	-3.424257	-3.716997	0.102824
C	6.0	5.745822	1.835287	2.725191
H	1.0	5.584687	0.774887	2.522881
H	1.0	5.921668	1.944343	3.800356
H	1.0	6.658659	2.141273	2.213897
C	6.0	4.914317	4.162479	2.559647
H	1.0	5.739080	4.490996	1.923592
H	1.0	5.231159	4.291099	3.600643

H	1.0	4.073352	4.835892	2.392860
C	6.0	-2.173905	-0.328117	-2.900368
H	1.0	-1.280410	-0.578377	-2.322840
H	1.0	-1.975559	-0.503677	-3.958523
H	1.0	-2.980470	-0.988395	-2.583217
C	6.0	3.613242	-1.530819	3.266768
H	1.0	3.963705	-0.513488	3.093901
H	1.0	4.019350	-1.876131	4.217792
H	1.0	2.523128	-1.511860	3.330568
C	6.0	-4.344748	3.649198	2.113109
H	1.0	-4.236182	4.459442	1.393350
H	1.0	-4.364175	4.089847	3.111112
H	1.0	-5.298353	3.146856	1.938810
C	6.0	5.115984	2.082230	-2.127810
H	1.0	4.406733	1.263722	-2.275150
H	1.0	5.982192	1.886286	-2.769266
H	1.0	4.655950	3.006690	-2.474975
C	6.0	-3.315197	1.634635	3.163139
H	1.0	-4.238635	1.061147	3.075606
H	1.0	-3.318795	2.153114	4.122345
H	1.0	-2.465482	0.948464	3.148279
C	6.0	-2.179235	4.514907	-1.078502
H	1.0	-1.198194	4.115385	-0.847089
C	6.0	1.941391	2.925506	-1.316113
H	1.0	2.561143	2.630971	-2.160631
H	1.0	1.222414	3.668063	-1.685422
H	1.0	1.370821	2.057529	-0.975380
C	6.0	5.594260	-2.429607	2.029396
H	1.0	5.944986	-3.120091	1.261664
H	1.0	6.039933	-2.724670	2.980898
H	1.0	5.939550	-1.424929	1.779094
C	6.0	-4.560331	4.186460	-1.262088
H	1.0	-5.423854	3.540362	-1.157841
C	6.0	3.344618	4.870860	-0.603386
H	1.0	3.937999	5.346280	0.180595
H	1.0	2.542398	5.566619	-0.874450
H	1.0	3.982321	4.755356	-1.480710
C	6.0	-5.079095	-2.283857	2.413002
C	6.0	5.377357	-3.549642	-1.378729
H	1.0	5.901727	-2.602047	-1.417784
C	6.0	3.182148	-2.117376	-3.465817

H	1.0	4.076090	-1.490089	-3.479762
H	1.0	2.700355	-2.051171	-4.442726
H	1.0	3.481550	-3.153242	-3.312474
C	6.0	1.657547	-0.262412	-2.808160
H	1.0	0.971081	0.107121	-2.043370
H	1.0	1.115537	-0.348673	-3.751443
H	1.0	2.467707	0.455548	-2.939278
B	5.0	-0.826547	-0.198577	0.739984
B	5.0	0.607257	-0.347153	0.882195
C	6.0	6.582168	3.304288	-0.529299
H	1.0	6.121840	4.278903	-0.704385
H	1.0	7.384299	3.182642	-1.265777
H	1.0	7.048754	3.327916	0.456300
C	6.0	-3.851743	-1.937271	3.277808
H	1.0	-3.198554	-2.793115	3.426910
H	1.0	-4.186832	-1.606248	4.266586
H	1.0	-3.240845	-1.140362	2.853298
C	6.0	-2.380661	-3.941856	1.209105
H	1.0	-1.832451	-3.027028	1.451992
H	1.0	-1.647484	-4.674634	0.852834
H	1.0	-2.825147	-4.347557	2.117532
C	6.0	-4.188895	-5.031987	-0.106076
H	1.0	-4.791766	-5.304461	0.763022
H	1.0	-3.472161	-5.843350	-0.274040
H	1.0	-4.850111	-4.999736	-0.972570
C	6.0	-6.130891	-2.151339	-0.607173
C	6.0	-3.592016	6.318390	-1.807711
H	1.0	-3.707725	7.342138	-2.138583
C	6.0	-2.619418	-3.393696	-1.164961
H	1.0	-3.240403	-3.215719	-2.041605
H	1.0	-1.964509	-4.242268	-1.394209
H	1.0	-1.976990	-2.524980	-1.002892
C	6.0	-2.329727	5.826874	-1.501884
H	1.0	-1.459568	6.463561	-1.594373
C	6.0	-4.705893	5.497296	-1.691187
H	1.0	-5.689794	5.878337	-1.930538
C	6.0	3.410626	-4.808645	-0.781766
H	1.0	2.421416	-4.843466	-0.342204
C	6.0	-7.254408	-3.123775	-0.227096
H	1.0	-6.913385	-4.161114	-0.202074
H	1.0	-8.060754	-3.060289	-0.966195

H	1.0	-7.688512	-2.890762	0.745587
C	6.0	1.026053	-2.598002	-2.246929
H	1.0	1.375653	-3.608292	-2.031123
H	1.0	0.449549	-2.635089	-3.174248
H	1.0	0.376834	-2.260478	-1.434803
C	6.0	-6.136619	-1.196933	2.664904
H	1.0	-5.832058	-0.228358	2.261418
H	1.0	-6.285753	-1.078349	3.743215
H	1.0	-7.102433	-1.454200	2.229946
C	6.0	-5.739580	-2.428695	-2.066364
H	1.0	-4.957242	-1.747691	-2.407342
H	1.0	-6.613248	-2.266835	-2.707198
H	1.0	-5.399820	-3.450109	-2.231096
C	6.0	-6.686938	-0.716299	-0.594872
H	1.0	-7.096173	-0.423664	0.369552
H	1.0	-7.494699	-0.639737	-1.331200
H	1.0	-5.913485	0.006783	-0.864231
C	6.0	-3.813533	1.464470	-3.484473
H	1.0	-4.644676	0.827171	-3.176341
H	1.0	-3.621487	1.295440	-4.545474
H	1.0	-4.102763	2.507664	-3.355385
C	6.0	-5.644696	-3.623098	2.903053
H	1.0	-6.525349	-3.939631	2.341432
H	1.0	-5.940498	-3.530469	3.954137
H	1.0	-4.904087	-4.421827	2.845849
C	6.0	3.997457	-5.958417	-1.289958
H	1.0	3.458469	-6.895895	-1.255060
C	6.0	5.963578	-4.702681	-1.877495
H	1.0	6.957876	-4.660115	-2.301906
C	6.0	5.271909	-5.906278	-1.838481
H	1.0	5.727146	-6.804692	-2.234054
C	6.0	-1.386038	2.017576	-3.096275
H	1.0	-1.659876	3.070994	-3.124723
H	1.0	-1.056145	1.730633	-4.096555
H	1.0	-0.553124	1.881325	-2.403664

(16) $B_2(Sil)_2$ initial model M06-2X-D3/Def2-TZVP

Si	14.0	-2.428785	-0.528208	-0.152585
Si	14.0	2.428810	0.528227	-0.152467
Si	14.0	-1.548473	-2.298374	-2.373214
Si	14.0	1.548610	2.298756	-2.372849

O	8.0	-2.657884	-1.587218	-1.386365
O	8.0	2.657975	1.587423	-1.386075
N	7.0	-3.530354	0.904465	-0.446006
N	7.0	-3.881507	-0.669845	0.950689
N	7.0	3.881464	0.669701	0.950913
N	7.0	3.530400	-0.904399	-0.446042
C	6.0	-4.293970	0.542198	0.579368
C	6.0	-4.244376	-1.524735	2.083946
C	6.0	4.293938	-0.542294	0.579448
C	6.0	4.244262	1.524429	2.084315
C	6.0	-3.366735	2.182310	-1.148770
C	6.0	-3.796744	-0.886213	3.401366
H	1.0	-4.332450	0.040307	3.610609
H	1.0	-2.729420	-0.663280	3.355130
H	1.0	-3.979078	-1.572851	4.229907
C	6.0	-5.742210	-1.833529	2.083365
H	1.0	-5.964352	-2.558638	2.867773
H	1.0	-6.035730	-2.262073	1.124225
H	1.0	-6.353039	-0.950744	2.268444
C	6.0	-2.489912	1.868211	-2.362257
H	1.0	-1.531777	1.454825	-2.035711
H	1.0	-2.297338	2.779436	-2.929970
H	1.0	-2.983537	1.144092	-3.013385
C	6.0	3.470406	2.827724	1.877099
H	1.0	3.691517	3.525840	2.684912
H	1.0	2.394737	2.635390	1.869141
H	1.0	3.749205	3.287709	0.927606
C	6.0	-3.470618	-2.828056	1.876523
H	1.0	-2.394935	-2.635807	1.868614
H	1.0	-3.749440	-3.287857	0.926948
H	1.0	-3.691798	-3.526291	2.684213
C	6.0	5.742072	1.833350	2.083809
H	1.0	6.035574	2.262062	1.124739
H	1.0	6.352976	0.950594	2.268772
H	1.0	5.964134	2.558363	2.868328
C	6.0	3.796662	0.885646	3.401617
H	1.0	3.978940	1.572150	4.230281
H	1.0	4.332435	-0.040874	3.610697
H	1.0	2.729355	0.662643	3.355332
C	6.0	2.489979	-1.867874	-2.362438
H	1.0	1.531838	-1.454550	-2.035831

H	1.0	2.297418	-2.779015	-2.930288
H	1.0	2.983595	-1.143649	-3.013457
C	6.0	-2.637096	3.182718	-0.249047
H	1.0	-3.227608	3.433166	0.634406
H	1.0	-2.443882	4.106125	-0.798534
H	1.0	-1.686759	2.753813	0.077283
B	5.0	-0.689686	-0.222425	0.460676
B	5.0	0.689679	0.222343	0.460659
C	6.0	3.366806	-2.182142	-1.148998
C	6.0	-4.710155	2.730524	-1.630150
H	1.0	-5.254738	1.966714	-2.187859
H	1.0	-4.532957	3.578043	-2.293767
H	1.0	-5.337528	3.082935	-0.812373
C	6.0	2.637181	-3.182692	-0.249423
H	1.0	1.686839	-2.753848	0.076974
H	1.0	3.227699	-3.433264	0.633991
H	1.0	2.443978	-4.106020	-0.799048
C	6.0	4.710232	-2.730268	-1.630462
H	1.0	5.254808	-1.966366	-2.188053
H	1.0	4.533041	-3.577685	-2.294212
H	1.0	5.337608	-3.082802	-0.812743
H	1.0	-0.813668	-1.287721	-3.162056
H	1.0	-0.591370	-3.128058	-1.614006
H	1.0	-2.309019	-3.166815	-3.296965
H	1.0	0.813804	1.288237	-3.161864
H	1.0	2.309202	3.167320	-3.296445
H	1.0	0.591504	3.128343	-1.613538
C	6.0	-5.376371	1.356321	1.206903
H	1.0	-5.080974	2.401661	1.247402
H	1.0	-6.280016	1.279823	0.599803
H	1.0	-5.597824	1.009639	2.209815
C	6.0	5.376256	-1.356538	1.206971
H	1.0	5.080831	-2.401878	1.247273
H	1.0	6.279969	-1.279962	0.599982
H	1.0	5.597602	-1.010022	2.209964

(17) $B_2(Sil)_2$ initial model M06-2X-D3/6-311+G(d)

Si	14.0	-2.429624	-0.510426	-0.127088
Si	14.0	2.429646	0.510432	-0.127034
Si	14.0	-1.584718	-2.317914	-2.383601
Si	14.0	1.584833	2.318280	-2.383296

O	8.0	-2.669726	-1.572853	-1.372812
O	8.0	2.669798	1.573035	-1.372597
N	7.0	-3.533784	0.933827	-0.434868
N	7.0	-3.909613	-0.640834	0.959267
N	7.0	3.909557	0.640704	0.959433
N	7.0	3.533842	-0.933760	-0.434956
C	6.0	-4.320810	0.570472	0.576711
C	6.0	-4.321587	-1.520014	2.059950
C	6.0	4.320793	-0.570545	0.576732
C	6.0	4.321419	1.519714	2.060294
C	6.0	-3.346700	2.218329	-1.125942
C	6.0	-3.946238	-0.901611	3.411459
H	1.0	-4.499403	0.018097	3.613544
H	1.0	-2.879343	-0.668252	3.427122
H	1.0	-4.162979	-1.603669	4.220748
C	6.0	-5.816993	-1.840308	1.974637
H	1.0	-6.080644	-2.577538	2.736835
H	1.0	-6.056876	-2.259491	0.995182
H	1.0	-6.447040	-0.964285	2.136130
C	6.0	-2.457977	1.902106	-2.333003
H	1.0	-1.505084	1.478608	-2.002449
H	1.0	-2.249394	2.814366	-2.896597
H	1.0	-2.949796	1.185260	-2.995545
C	6.0	3.525888	2.814585	1.866855
H	1.0	3.783014	3.534944	2.646197
H	1.0	2.451133	2.619434	1.920729
H	1.0	3.748372	3.258216	0.893582
C	6.0	-3.526038	-2.814856	1.866389
H	1.0	-2.451289	-2.619717	1.920410
H	1.0	-3.748417	-3.258328	0.893020
H	1.0	-3.783252	-3.535342	2.645586
C	6.0	5.816833	1.840023	1.975184
H	1.0	6.056822	2.259342	0.995814
H	1.0	6.446865	0.963980	2.136627
H	1.0	6.080400	2.577149	2.737512
C	6.0	3.945934	0.901096	3.411667
H	1.0	4.162607	1.603018	4.221092
H	1.0	4.499069	-0.018652	3.613650
H	1.0	2.879035	0.667745	3.427190
C	6.0	2.458136	-1.901786	-2.333278
H	1.0	1.505221	-1.478354	-2.002703

H	1.0	2.249594	-2.813969	-2.897011
H	1.0	2.949967	-1.184836	-2.995699
C	6.0	-2.617450	3.204983	-0.207425
H	1.0	-3.215347	3.452802	0.674015
H	1.0	-2.408728	4.135321	-0.742787
H	1.0	-1.672965	2.768498	0.127728
B	5.0	-0.690285	-0.220014	0.494459
B	5.0	0.690278	0.219897	0.494387
C	6.0	3.346815	-2.218165	-1.126225
C	6.0	-4.680107	2.784823	-1.621350
H	1.0	-5.229993	2.030562	-2.189449
H	1.0	-4.488925	3.634143	-2.281344
H	1.0	-5.315422	3.144013	-0.810629
C	6.0	2.617544	-3.204970	-0.207886
H	1.0	1.673035	-2.768555	0.127290
H	1.0	3.215404	-3.452910	0.673544
H	1.0	2.408866	-4.135232	-0.743398
C	6.0	4.680257	-2.784555	-1.621658
H	1.0	5.230146	-2.030198	-2.189629
H	1.0	4.489126	-3.633788	-2.281778
H	1.0	5.315548	-3.143838	-0.810960
H	1.0	-0.836809	-1.321548	-3.181180
H	1.0	-0.635186	-3.167656	-1.633590
H	1.0	-2.380541	-3.168601	-3.294769
H	1.0	0.836915	1.322053	-3.181039
H	1.0	2.380697	3.169093	-3.294310
H	1.0	0.635302	3.167926	-1.633173
C	6.0	-5.424984	1.381137	1.176907
H	1.0	-5.123716	2.424158	1.258521
H	1.0	-6.304630	1.331162	0.531015
H	1.0	-5.695213	1.015938	2.162631
C	6.0	5.424933	-1.381285	1.176887
H	1.0	5.123692	-2.424330	1.258283
H	1.0	6.304639	-1.331162	0.531089
H	1.0	5.695055	-1.016256	2.162703

(18) $B_2(Sil)_2$ initial model B3LYP-D3/6-311+G(d)

Si	14.0	-2.429144	-0.510786	-0.128937
Si	14.0	2.429142	0.510795	-0.128779
Si	14.0	-1.589306	-2.313736	-2.390810
Si	14.0	1.589435	2.314138	-2.390389

O	8.0	-2.672300	-1.569480	-1.377353
O	8.0	2.672370	1.569702	-1.377001
N	7.0	-3.530655	0.936354	-0.431785
N	7.0	-3.908201	-0.641353	0.958373
N	7.0	3.908132	0.641187	0.958638
N	7.0	3.530679	-0.936288	-0.431808
C	6.0	-4.317067	0.572127	0.579827
C	6.0	-4.318741	-1.521448	2.058852
C	6.0	4.317036	-0.572221	0.579906
C	6.0	4.318646	1.521132	2.059247
C	6.0	-3.343437	2.221262	-1.122068
C	6.0	-3.934358	-0.907685	3.409958
H	1.0	-4.482546	0.014054	3.616411
H	1.0	-2.866399	-0.678895	3.420978
H	1.0	-4.150338	-1.610415	4.218873
C	6.0	-5.815834	-1.835220	1.979920
H	1.0	-6.078894	-2.573542	2.741256
H	1.0	-6.062278	-2.250554	1.000444
H	1.0	-6.441322	-0.957007	2.147099
C	6.0	-2.455254	1.905886	-2.329773
H	1.0	-1.502057	1.482533	-1.999973
H	1.0	-2.247310	2.818558	-2.892929
H	1.0	-2.947274	1.189299	-2.992459
C	6.0	3.529471	2.818879	1.859150
H	1.0	3.786166	3.540039	2.637890
H	1.0	2.453668	2.628384	1.908865
H	1.0	3.758039	3.259144	0.885761
C	6.0	-3.529597	-2.819186	1.858568
H	1.0	-2.453790	-2.628726	1.908326
H	1.0	-3.758164	-3.259295	0.885108
H	1.0	-3.786321	-3.540457	2.637195
C	6.0	5.815732	1.834952	1.980366
H	1.0	6.062170	2.250432	1.000950
H	1.0	6.441241	0.956731	2.147423
H	1.0	6.078770	2.573173	2.741807
C	6.0	3.934274	0.907162	3.410262
H	1.0	4.150239	1.609777	4.219280
H	1.0	4.482480	-0.014596	3.616578
H	1.0	2.866321	0.678349	3.421247
C	6.0	2.455369	-1.905542	-2.329989
H	1.0	1.502161	-1.482217	-2.000181

H	1.0	2.247437	-2.818134	-2.893280
H	1.0	2.947437	-1.188875	-2.992551
C	6.0	-2.613691	3.207059	-0.203026
H	1.0	-3.211359	3.454435	0.678699
H	1.0	-2.404590	4.137676	-0.737741
H	1.0	-1.669402	2.769779	0.131668
B	5.0	-0.688803	-0.224844	0.492302
B	5.0	0.688764	0.224738	0.492307
C	6.0	3.343482	-2.221098	-1.122280
C	6.0	-4.677012	2.787933	-1.616791
H	1.0	-5.226083	2.034299	-2.186512
H	1.0	-4.486231	3.638643	-2.275106
H	1.0	-5.313080	3.144978	-0.805713
C	6.0	2.613676	-3.207014	-0.203413
H	1.0	1.669372	-2.769773	0.131289
H	1.0	3.211294	-3.454515	0.678311
H	1.0	2.404597	-4.137556	-0.738267
C	6.0	4.677076	-2.787721	-1.617009
H	1.0	5.226195	-2.034014	-2.186587
H	1.0	4.486317	-3.638330	-2.275460
H	1.0	5.313087	-3.144898	-0.805945
H	1.0	-0.838072	-1.316589	-3.184291
H	1.0	-0.642501	-3.169064	-1.643621
H	1.0	-2.387186	-3.158766	-3.305423
H	1.0	0.838223	1.317131	-3.184067
H	1.0	2.387369	3.159307	-3.304826
H	1.0	0.642607	3.169355	-1.643103
C	6.0	-5.418439	1.383502	1.184174
H	1.0	-5.118380	2.427184	1.260367
H	1.0	-6.302284	1.329682	0.544275
H	1.0	-5.681661	1.021103	2.172891
C	6.0	5.418392	-1.383682	1.184166
H	1.0	5.118307	-2.427367	1.260248
H	1.0	6.302235	-1.329818	0.544271
H	1.0	5.681623	-1.021397	2.172922

(19) $B_2(Sil)_2$ final model M06-2X-D3/6-311+G(d)

Si	14.0	-2.429624	-0.510426	-0.127088
Si	14.0	2.429646	0.510432	-0.127034
O	8.0	-2.669726	-1.572853	-1.372812
O	8.0	2.669798	1.573035	-1.372597

N	7.0	-3.533784	0.933827	-0.434868
N	7.0	-3.909613	-0.640834	0.959267
N	7.0	3.909557	0.640704	0.959433
N	7.0	3.533842	-0.933760	-0.434956
C	6.0	-4.320810	0.570472	0.576711
C	6.0	4.320793	-0.570545	0.576732
B	5.0	-0.690285	-0.220014	0.494459
B	5.0	0.690278	0.219897	0.494387
H	1.0	-5.108940	1.161400	1.034035
H	1.0	-4.266023	-1.136611	1.759102
H	1.0	-3.493413	1.866427	-0.812168
H	1.0	-1.854121	-1.951617	-1.713788
H	1.0	1.854214	1.951860	-1.713551
H	1.0	4.265887	1.136356	1.759381
H	1.0	5.108893	-1.161533	1.034030
H	1.0	3.493512	-1.866304	-0.812399

(20) $B_2(Sil^R)_2$-SiH_2 M06-2X-D3/ Def2-TZVP

Si	14.0	-2.553486	0.321523	0.205353
Si	14.0	2.479377	-0.447305	0.245163
Si	14.0	-4.426725	-2.265857	0.552897
Si	14.0	-0.024028	-0.165698	2.594799
H	1.0	-0.168111	-1.354755	3.491332
Si	14.0	4.087073	2.319187	0.603824
H	1.0	0.159871	1.016440	3.495577
O	8.0	-3.739990	-0.780249	0.312349
O	8.0	3.536114	0.770425	0.444537
N	7.0	-3.236894	1.981739	0.619742
N	7.0	2.955004	-1.333068	-1.319729
N	7.0	3.359737	-2.005017	0.683841
N	7.0	-2.828539	1.264138	-1.361881
C	6.0	3.393764	-2.363726	-0.590534
C	6.0	-3.134352	2.347494	-0.656017
C	6.0	-5.068593	-2.260949	2.359296
C	6.0	3.654800	-2.761790	1.914038
C	6.0	-3.351980	2.804634	1.839708
C	6.0	2.355850	-1.374406	-2.668579
C	6.0	3.856498	-3.672963	-1.113974
C	6.0	-2.450728	1.114329	-2.774724
C	6.0	-5.852527	-2.307241	-0.741475
C	6.0	-3.277994	3.724820	-1.187818

C	6.0	-3.105220	-3.623484	0.259333
C	6.0	-2.148636	-3.721437	1.459281
H	1.0	-1.709252	-2.754497	1.719922
H	1.0	-1.323891	-4.393456	1.195474
H	1.0	-2.634961	-4.139529	2.340150
C	6.0	2.465147	-3.665509	2.247560
H	1.0	1.546018	-3.075880	2.274515
H	1.0	2.615097	-4.125214	3.226073
H	1.0	2.359776	-4.462987	1.512295
C	6.0	-3.958792	-1.773482	3.310186
H	1.0	-3.231766	-2.549759	3.534483
H	1.0	-4.406736	-1.462359	4.259912
H	1.0	-3.403888	-0.924840	2.911502
C	6.0	-6.517210	-0.922339	-0.832786
H	1.0	-7.023763	-0.629729	0.084244
H	1.0	-7.266618	-0.937410	-1.631996
H	1.0	-5.782831	-0.149557	-1.071752
C	6.0	-2.018410	3.493573	2.138777
H	1.0	-1.210474	2.758605	2.138495
H	1.0	-2.060127	3.964926	3.122485
H	1.0	-1.800680	4.268870	1.404689
C	6.0	2.672686	3.530619	0.135557
C	6.0	-6.236568	-1.268989	2.481636
H	1.0	-5.980937	-0.285864	2.080710
H	1.0	-6.493073	-1.141929	3.538396
H	1.0	-7.132791	-1.619531	1.969847
C	6.0	-5.554517	-3.630896	2.850161
H	1.0	-6.348741	-4.045744	2.226665
H	1.0	-5.951895	-3.531513	3.866429
H	1.0	-4.743453	-4.358825	2.891605
C	6.0	1.821383	0.029431	-2.936389
H	1.0	1.094251	0.310928	-2.172382
H	1.0	1.329724	0.059717	-3.909930
H	1.0	2.630389	0.759079	-2.935259
C	6.0	-3.665252	1.837798	2.978013
H	1.0	-4.570957	1.267862	2.767541
H	1.0	-3.810967	2.395180	3.903674
H	1.0	-2.833187	1.146960	3.123267
C	6.0	4.946140	-3.571456	1.795226
H	1.0	4.845852	-4.425257	1.126934
H	1.0	5.210737	-3.951148	2.782944

H	1.0	5.763952	-2.940429	1.440987
C	6.0	-5.315875	-2.601286	-2.149889
H	1.0	-4.560864	-1.872211	-2.450307
H	1.0	-6.139683	-2.530315	-2.868606
H	1.0	-4.886678	-3.597757	-2.243947
C	6.0	3.809261	-1.727912	3.025367
H	1.0	4.607478	-1.024194	2.789352
H	1.0	4.049760	-2.228072	3.963756
H	1.0	2.876438	-1.177332	3.158441
C	6.0	3.039014	-4.796111	-1.078130
H	1.0	2.027500	-4.713150	-0.700714
C	6.0	-2.222075	-3.290272	-0.952653
H	1.0	-2.777787	-3.201948	-1.885098
H	1.0	-1.488982	-4.095000	-1.085694
H	1.0	-1.658690	-2.369636	-0.782871
C	6.0	-4.481624	3.827618	1.719796
H	1.0	-4.245940	4.626676	1.018488
H	1.0	-4.648271	4.281942	2.697392
H	1.0	-5.407423	3.341882	1.404800
C	6.0	5.151628	-3.766485	-1.613190
H	1.0	5.777657	-2.882593	-1.639105
C	6.0	-3.745252	-5.003042	0.045836
H	1.0	-4.403720	-5.288486	0.868889
H	1.0	-2.955945	-5.760177	-0.018741
H	1.0	-4.323034	-5.058315	-0.877433
C	6.0	4.627084	2.529246	2.430737
C	6.0	-4.500596	4.147338	-1.695385
H	1.0	-5.342218	3.465172	-1.702367
C	6.0	-3.681653	1.267764	-3.667385
H	1.0	-4.457333	0.560520	-3.368219
H	1.0	-3.412679	1.070089	-4.706620
H	1.0	-4.085436	2.278870	-3.608757
C	6.0	-1.897738	-0.302761	-2.897216
H	1.0	-1.031700	-0.430418	-2.244721
H	1.0	-1.600935	-0.503673	-3.927480
H	1.0	-2.651521	-1.033276	-2.604867
B	5.0	0.698369	-0.266943	0.784799
B	5.0	-0.818877	-0.038721	0.824330
C	6.0	-6.925469	-3.354936	-0.419121
H	1.0	-6.508608	-4.360519	-0.330009
H	1.0	-7.669369	-3.377482	-1.223231

H	1.0	-7.456002	-3.129894	0.506203
C	6.0	3.539971	1.970294	3.365076
H	1.0	2.646962	2.589191	3.396432
H	1.0	3.936199	1.915347	4.384835
H	1.0	3.222838	0.969119	3.075343
C	6.0	1.637043	3.641505	1.266252
H	1.0	1.254575	2.661764	1.567818
H	1.0	0.783566	4.226361	0.901475
H	1.0	2.025104	4.157724	2.143486
C	6.0	3.204895	4.937679	-0.171855
H	1.0	3.799465	5.349446	0.645932
H	1.0	2.358539	5.614889	-0.334169
H	1.0	3.816001	4.962399	-1.074945
C	6.0	5.574093	2.390796	-0.616740
C	6.0	4.817571	-6.111098	-2.023137
H	1.0	5.193065	-7.063229	-2.374261
C	6.0	1.887496	3.021169	-1.082388
H	1.0	2.504469	2.833346	-1.958887
H	1.0	1.147650	3.780842	-1.365320
H	1.0	1.338506	2.110408	-0.830735
C	6.0	3.521175	-6.013872	-1.536710
H	1.0	2.882786	-6.887028	-1.511627
C	6.0	5.631714	-4.986263	-2.063172
H	1.0	6.641585	-5.059099	-2.444488
C	6.0	-2.190786	4.591590	-1.166245
H	1.0	-1.236601	4.241467	-0.789569
C	6.0	6.513819	3.576080	-0.363148
H	1.0	5.993938	4.535043	-0.411400
H	1.0	7.300012	3.590221	-1.126236
H	1.0	7.006585	3.512367	0.607185
C	6.0	-1.356803	2.103178	-3.186391
H	1.0	-1.737415	3.118230	-3.287592
H	1.0	-0.946323	1.802832	-4.152197
H	1.0	-0.548088	2.096718	-2.451910
C	6.0	5.907823	1.726098	2.706738
H	1.0	5.815274	0.685314	2.392868
H	1.0	6.109125	1.731079	3.782988
H	1.0	6.780336	2.151614	2.211038
C	6.0	5.084461	2.473663	-2.069443
H	1.0	4.429497	1.633721	-2.310465
H	1.0	5.946890	2.419153	-2.742775

H	1.0	4.556606	3.400642	-2.290477
C	6.0	6.383123	1.084155	-0.530236
H	1.0	6.870121	0.944820	0.432249
H	1.0	7.167291	1.099507	-1.295361
H	1.0	5.745943	0.216386	-0.717568
C	6.0	3.395362	-1.729972	-3.730115
H	1.0	4.259171	-1.067028	-3.650356
H	1.0	2.955341	-1.606761	-4.721326
H	1.0	3.733497	-2.761314	-3.639662
C	6.0	4.901096	3.988975	2.817508
H	1.0	5.659306	4.455245	2.184822
H	1.0	5.266141	4.026839	3.849804
H	1.0	4.001211	4.602386	2.771639
C	6.0	-2.331836	5.884980	-1.644730
H	1.0	-1.484623	6.558006	-1.629446
C	6.0	-4.637920	5.443219	-2.171651
H	1.0	-5.591425	5.774478	-2.561061
C	6.0	-3.555781	6.312519	-2.143686
H	1.0	-3.665201	7.323552	-2.513406
C	6.0	1.180398	-2.356590	-2.698288
H	1.0	1.523023	-3.389486	-2.649501
H	1.0	0.616439	-2.236548	-3.625474
H	1.0	0.512780	-2.160318	-1.855421

(21) $B_2(SiI)_2$-SiH_2 initial model M06-2X-D3/ Def2-TZVP

Si	14.0	0.000186	-2.448984	0.002473
H	1.0	0.427101	-3.343943	1.121569
H	1.0	-0.427245	-3.345614	-1.115102
Si	14.0	2.459910	-0.248344	-0.811302
Si	14.0	-2.459445	-0.248157	0.811635
Si	14.0	2.341497	-1.942375	-3.267698
Si	14.0	-2.342111	-1.939987	3.269678
O	8.0	2.976709	-0.801083	-2.261220
O	8.0	-2.976940	-0.799800	2.261724
N	7.0	3.807930	-0.455262	0.399845
N	7.0	-3.244362	1.388585	0.517440
N	7.0	-3.806381	-0.457179	-0.400405
N	7.0	3.243415	1.389317	-0.518878
C	6.0	-3.958117	0.860386	-0.479368
C	6.0	3.958397	0.862489	0.477828
C	6.0	-4.287852	-1.560541	-1.240512

C	6.0	4.291036	-1.557627	1.240312
C	6.0	-2.769495	2.760221	0.734603
C	6.0	2.766245	2.760197	-0.735982
C	6.0	-3.409765	-1.670955	-2.488719
H	1.0	-2.365021	-1.799521	-2.198008
H	1.0	-3.718858	-2.528406	-3.089088
H	1.0	-3.487556	-0.775260	-3.108132
C	6.0	3.414476	-1.667560	2.489636
H	1.0	2.369476	-1.797014	2.200238
H	1.0	3.724795	-2.524348	3.090319
H	1.0	3.492421	-0.771350	3.108274
C	6.0	-1.920940	2.700630	2.006679
H	1.0	-1.070779	2.028240	1.867255
H	1.0	-1.541003	3.693756	2.249222
H	1.0	-2.518420	2.337473	2.844503
C	6.0	4.136732	-2.823639	0.395796
H	1.0	4.733058	-2.749346	-0.515529
H	1.0	4.468695	-3.692214	0.965249
H	1.0	3.090532	-2.974893	0.120645
C	6.0	-5.762453	-1.390136	-1.608278
H	1.0	-5.926902	-0.601530	-2.340767
H	1.0	-6.126597	-2.320085	-2.046350
H	1.0	-6.357373	-1.177938	-0.717995
C	6.0	-4.133878	-2.825769	-0.394760
H	1.0	-4.731439	-2.751074	0.515724
H	1.0	-4.464589	-3.695007	-0.963931
H	1.0	-3.087961	-2.976187	-0.118122
C	6.0	5.765965	-1.385985	1.606145
H	1.0	5.930819	-0.596404	2.337496
H	1.0	6.131173	-2.315204	2.044880
H	1.0	6.359697	-1.174470	0.714909
C	6.0	3.930753	3.722634	-0.970416
H	1.0	4.549601	3.365747	-1.794516
H	1.0	3.541478	4.707812	-1.232246
H	1.0	4.564002	3.841305	-0.092443
C	6.0	1.916687	2.698973	-2.007313
H	1.0	1.067678	2.025309	-1.866984
H	1.0	1.535004	3.691455	-2.249761
H	1.0	2.514007	2.336557	-2.845575
B	5.0	-0.718003	-0.645194	0.260476
B	5.0	0.719092	-0.646128	-0.258587

C	6.0	1.877043	3.205999	0.429051
H	1.0	2.437763	3.314276	1.358252
H	1.0	1.415940	4.169378	0.202308
H	1.0	1.090896	2.463665	0.586288
C	6.0	-3.935693	3.720920	0.967815
H	1.0	-4.554683	3.363267	1.791475
H	1.0	-3.548175	4.706766	1.229738
H	1.0	-4.568383	3.838394	0.089278
C	6.0	-1.880012	3.207160	-0.429774
H	1.0	-2.440087	3.314244	-1.359500
H	1.0	-1.420696	4.171362	-0.202902
H	1.0	-1.092502	2.466097	-0.586111
H	1.0	0.904995	-1.707854	-3.506805
H	1.0	3.074331	-1.827740	-4.546603
H	1.0	2.554065	-3.292808	-2.709973
H	1.0	-3.075988	-1.824473	4.547914
H	1.0	-2.553771	-3.290995	2.713004
H	1.0	-0.905885	-1.704745	3.509733
C	6.0	-4.739754	1.595838	-1.517236
H	1.0	-5.801524	1.543929	-1.271841
H	1.0	-4.591284	1.134482	-2.490850
H	1.0	-4.444381	2.636961	-1.567512
C	6.0	4.740101	1.599421	1.514588
H	1.0	5.801662	1.548969	1.267963
H	1.0	4.593428	1.138206	2.488533
H	1.0	4.443297	2.640142	1.564820

(22) $B_2(Sil)_2$-SiH_2 initial model   M06-2X-D3/6-311+G(d)

Si	14.0	0.00000000	0.00000000	0.00000000
H	1.0	1.20070190	0.89645001	0.00000000
H	1.0	-1.20070190	0.89648693	0.00000000
Si	14.0	0.15082397	-2.23005015	2.58229433
Si	14.0	-0.15377487	-2.22814479	-2.58257055
Si	14.0	-2.19693013	-0.53921074	3.46337047
Si	14.0	2.19106015	-0.53456784	-3.46629842
O	8.0	-1.00319813	-1.68464123	3.62361770
O	8.0	0.99801170	-1.68086248	-3.62537822
N	7.0	1.78295199	-2.02780522	3.39623330
N	7.0	-0.73237762	-3.87651210	-3.19311679
N	7.0	-1.78762097	-2.02580268	-3.39310693
N	7.0	0.72937854	-3.87893992	3.19131565

C	6.0	-1.92336973	-3.34539343	-3.49641518
C	6.0	1.91946093	-3.34744829	3.49766835
C	6.0	-2.73404871	-0.91259817	-3.56051655
C	6.0	2.72814431	-0.91413744	3.56740933
C	6.0	-0.36715113	-5.24632607	-2.80017378
C	6.0	0.36617087	-5.24819070	2.79443682
C	6.0	-3.59265294	-0.77970803	-2.29852173
H	1.0	-2.95316307	-0.64665006	-1.42244229
H	1.0	-4.25777668	0.08368805	-2.38235993
H	1.0	-4.20973452	-1.66821941	-2.13983047
C	6.0	3.58980717	-0.77899772	2.30774272
H	1.0	2.95238630	-0.64528399	1.43025326
H	1.0	4.25405218	0.08480102	2.39433824
H	1.0	4.20798152	-1.66680645	2.14942415
C	6.0	1.13066856	-5.19158861	-2.48104360
H	1.0	1.32528780	-4.50253797	-1.65435948
H	1.0	1.49133942	-6.18160539	-2.19309275
H	1.0	1.69530828	-4.85396346	-3.35326112
C	6.0	1.86554319	0.34073128	3.73463154
H	1.0	1.21846847	0.24848101	4.61045729
H	1.0	2.50254241	1.21878086	3.86008352
H	1.0	1.23988977	0.50088097	2.85250556
C	6.0	-3.59745503	-1.08984017	-4.81294798
H	1.0	-4.34507679	-1.87656811	-4.70773085
H	1.0	-4.13584577	-0.16049243	-5.01093851
H	1.0	-2.97393268	-1.30991428	-5.68315080
C	6.0	-1.87280308	0.34311351	-3.72839135
H	1.0	-1.22808840	0.25251741	-4.60612985
H	1.0	-2.51077442	1.22087558	-3.85088902
H	1.0	-1.24483886	0.50252981	-2.84778701
C	6.0	3.58846868	-1.09240737	4.82180571
H	1.0	4.33660009	-1.87878928	4.71761354
H	1.0	4.12604805	-0.16309998	5.02217943
H	1.0	2.96281876	-1.31367058	5.69017545
C	6.0	0.58451404	-6.23410903	3.94533438
H	1.0	0.03906158	-5.90362985	4.83168072
H	1.0	0.21098552	-7.22091774	3.66094641
H	1.0	1.63507859	-6.34823154	4.21521359
C	6.0	-1.13110079	-5.19407035	2.47262452
H	1.0	-1.32483905	-4.50303912	1.64738399
H	1.0	-1.49024587	-6.18368167	2.18137556

H	1.0	-1.69772189	-4.85931487	3.34466680
B	5.0	-0.01504482	-1.81111623	-0.76348615
B	5.0	0.01484884	-1.81092486	0.76346633
C	6.0	1.12925422	-5.65860864	1.52838726
H	1.0	2.20254498	-5.76025547	1.70463597
H	1.0	0.76205451	-6.61972227	1.15798804
H	1.0	0.98119564	-4.90254076	0.75278565
C	6.0	-0.58675148	-6.22967801	-3.95303804
H	1.0	-0.04320711	-5.89658050	-4.83957557
H	1.0	-0.21186120	-7.21685165	-3.67172460
H	1.0	-1.63771194	-6.34403675	-4.22127908
C	6.0	-1.12745387	-5.66053122	-1.53369304
H	1.0	-2.20099658	-5.76265259	-1.70810198
H	1.0	-0.75872004	-6.62224176	-1.16637738
H	1.0	-0.97852012	-4.90627623	-0.75651457
H	1.0	-2.98069663	-0.75532299	2.23065056
H	1.0	-3.07965178	-0.68636019	4.64065715
H	1.0	-1.60539093	0.81519390	3.46394770
H	1.0	3.07170081	-0.67990462	-4.64537250
H	1.0	1.59838837	0.81934283	-3.46454990
H	1.0	2.97732715	-0.75116574	-2.23526968
C	6.0	-3.17879616	-4.07988707	-3.84544976
H	1.0	-3.31869666	-4.06559500	-4.92878383
H	1.0	-4.03890972	-3.59799134	-3.38381534
H	1.0	-3.13710765	-5.11337494	-3.51756594
C	6.0	3.17472481	-4.08161716	3.84794792
H	1.0	3.31189522	-4.06989690	4.93166817
H	1.0	4.03554639	-3.59781729	3.38965844
H	1.0	3.13479057	-5.11433553	3.51742032

(23) $B_2(Sil)_2$-SiH_2 initial model B3LYP-D3/6-311+G(d)

Si	14.0	0.000792	-2.687730	0.003088
H	1.0	0.411155	-3.585465	1.133962
H	1.0	-0.409290	-3.587991	-1.125881
Si	14.0	2.443503	-0.325636	-0.748232
Si	14.0	-2.443256	-0.325584	0.748729
Si	14.0	2.690740	-1.921122	-3.372404
Si	14.0	-2.691878	-1.918752	3.374189
O	8.0	3.078731	-0.810454	-2.200452
O	8.0	-3.079344	-0.809249	2.200957
N	7.0	3.817713	-0.427617	0.492705

N	7.0	-3.135552	1.375599	0.442053
N	7.0	-3.816410	-0.429550	-0.493256
N	7.0	3.134837	1.376276	-0.443564
C	6.0	-3.919681	0.904566	-0.536374
C	6.0	3.920002	0.906595	0.534716
C	6.0	-4.323506	-1.486943	-1.394170
C	6.0	4.326714	-1.484040	1.393668
C	6.0	-2.729932	2.745619	0.802782
C	6.0	2.726766	2.745645	-0.804067
C	6.0	-3.486183	-1.511368	-2.685300
H	1.0	-2.428896	-1.644173	-2.446476
H	1.0	-3.803946	-2.333968	-3.332343
H	1.0	-3.592491	-0.581849	-3.251969
C	6.0	3.490826	-1.508399	2.685728
H	1.0	2.433402	-1.642308	2.448138
H	1.0	3.810025	-2.330316	3.332932
H	1.0	3.596921	-0.578434	3.251701
C	6.0	-1.819074	2.602948	2.034617
H	1.0	-0.948234	1.982416	1.807901
H	1.0	-1.465131	3.584502	2.359185
H	1.0	-2.358806	2.140657	2.864426
C	6.0	4.143726	-2.812513	0.641173
H	1.0	4.711781	-2.811050	-0.292590
H	1.0	4.495080	-3.643896	1.257083
H	1.0	3.092186	-2.990670	0.406427
C	6.0	-5.819140	-1.301713	-1.701287
H	1.0	-6.019571	-0.450001	-2.352139
H	1.0	-6.197099	-2.189757	-2.213854
H	1.0	-6.391676	-1.177055	-0.778389
C	6.0	-4.140269	-2.814816	-0.640682
H	1.0	-4.709347	-2.813246	0.292460
H	1.0	-4.490292	-3.646849	-1.256473
H	1.0	-3.088851	-2.992003	-0.404680
C	6.0	5.822526	-1.297375	1.699022
H	1.0	6.022954	-0.445087	2.349122
H	1.0	6.201794	-2.184782	2.211725
H	1.0	6.393942	-1.172811	0.775417
C	6.0	3.946732	3.599393	-1.189149
H	1.0	4.509591	3.116744	-1.991471
H	1.0	3.621305	4.581671	-1.542792
H	1.0	4.626622	3.763166	-0.351163

C	6.0	1.815036	2.601421	-2.035078
H	1.0	0.945287	1.979676	-1.807478
H	1.0	1.459409	3.582407	-2.359527
H	1.0	2.354719	2.139758	-2.865273
B	5.0	-0.726283	-0.876315	0.248052
B	5.0	0.727150	-0.876629	-0.245740
C	6.0	1.921220	3.392637	0.338263
H	1.0	2.528334	3.565596	1.229363
H	1.0	1.518828	4.359352	0.022566
H	1.0	1.087984	2.746802	0.619463
C	6.0	-3.951578	3.597494	1.186704
H	1.0	-4.514416	3.114013	1.988539
H	1.0	-3.628009	4.580299	1.540585
H	1.0	-4.630944	3.760150	0.348079
C	6.0	-1.924419	3.393813	-0.338887
H	1.0	-2.530985	3.565598	-1.230586
H	1.0	-1.523999	4.361268	-0.022943
H	1.0	-1.089788	2.749414	-0.619215
H	1.0	1.276165	-1.790076	-3.793562
H	1.0	3.573611	-1.637877	-4.532281
H	1.0	2.949939	-3.297464	-2.886355
H	1.0	-3.575991	-1.634949	4.532989
H	1.0	-2.949936	-3.295636	2.889080
H	1.0	-1.277779	-1.786622	3.796593
C	6.0	-4.727076	1.704421	-1.510961
H	1.0	-5.785472	1.652164	-1.245017
H	1.0	-4.616282	1.305562	-2.518578
H	1.0	-4.429495	2.747876	-1.517324
C	6.0	4.727474	1.707825	1.508104
H	1.0	5.785672	1.656500	1.241187
H	1.0	4.618031	1.309440	2.516053
H	1.0	4.428847	2.750984	1.514127

(24) $B_2(Sil)_2$-SiH_2 final model M06-2X-D3/6-311+G(d)

Si	14.0	0.00000000	0.00000000	0.00000000
H	1.0	1.20070190	0.89645001	0.00000000
H	1.0	-1.20070190	0.89648693	0.00000000
Si	14.0	0.15082397	-2.23005015	2.58229433
Si	14.0	-0.15377487	-2.22814479	-2.58257055
O	8.0	-1.00319813	-1.68464123	3.62361770
O	8.0	0.99801170	-1.68086248	-3.62537822

N	7.0	1.78295199	-2.02780522	3.39623330
N	7.0	-0.73237762	-3.87651210	-3.19311679
N	7.0	-1.78762097	-2.02580268	-3.39310693
N	7.0	0.72937854	-3.87893992	3.19131565
C	6.0	-1.92336973	-3.34539343	-3.49641518
C	6.0	1.91946093	-3.34744829	3.49766835
B	5.0	-0.01504482	-1.81111623	-0.76348615
B	5.0	0.01484884	-1.81092486	0.76346633
H	1.0	2.55143233	-1.37709793	3.41424174
H	1.0	-1.63567780	-1.07463176	3.23308948
H	1.0	2.83059385	-3.88958973	3.73316975
H	1.0	0.56873894	-4.86565323	3.07503672
H	1.0	1.63036855	-1.07033810	-3.23553399
H	1.0	-2.55662886	-1.37564900	-3.40844846
H	1.0	-2.83453741	-3.88781095	-3.73114620
H	1.0	-0.57072374	-4.86331885	-3.07906612

(25)$B_2(Sil^R)_2$-$SiCl_2$ M06-2X-D3/ Def2-TZVP

Si	14.0	-2.502694	0.430254	0.052587
Si	14.0	2.542153	-0.304601	0.004773
Si	14.0	-4.076858	-2.368402	0.353033
Si	14.0	0.045789	0.338714	2.401477
Cl	17.0	-0.149928	-1.155796	3.896300
Si	14.0	4.489993	2.252993	0.252149
Cl	17.0	0.199869	2.050847	3.643822
O	8.0	-3.519112	-0.818349	0.215663
O	8.0	3.722029	0.800990	0.035135
N	7.0	-3.435988	1.953111	0.485825
N	7.0	2.775806	-1.302572	-1.526840
N	7.0	3.223580	-1.949351	0.470894
N	7.0	-2.943183	1.315164	-1.510248
C	6.0	3.111872	-2.357604	-0.791704
C	6.0	-3.455364	2.319694	-0.784428
C	6.0	-4.670349	-2.570144	2.160154
C	6.0	3.323096	-2.745019	1.714008
C	6.0	-3.869443	2.657112	1.708908
C	6.0	2.456548	-1.181965	-2.956196
C	6.0	3.290461	-3.744361	-1.287852
C	6.0	-2.255067	1.409372	-2.814044
C	6.0	-5.516501	-2.432229	-0.922115
C	6.0	-3.986214	3.592845	-1.330096

C	6.0	-2.637923	-3.562723	-0.072130
C	6.0	-1.652611	-3.683318	1.101472
H	1.0	-1.299558	-2.707535	1.447805
H	1.0	-0.774245	-4.247906	0.764800
H	1.0	-2.072765	-4.223489	1.948885
C	6.0	2.010434	-3.488626	1.968305
H	1.0	1.168373	-2.798018	1.896985
H	1.0	2.014692	-3.912069	2.974119
H	1.0	1.873983	-4.304936	1.259119
C	6.0	-3.594044	-2.033030	3.123205
H	1.0	-2.769840	-2.728366	3.258392
H	1.0	-4.039165	-1.864385	4.109272
H	1.0	-3.156857	-1.089619	2.793466
C	6.0	-6.331828	-1.128135	-0.859884
H	1.0	-6.862675	-0.999816	0.080621
H	1.0	-7.080227	-1.137580	-1.660077
H	1.0	-5.689106	-0.256966	-1.010129
C	6.0	-2.885325	3.783179	2.031090
H	1.0	-1.864081	3.397397	2.041214
H	1.0	-3.105805	4.187355	3.020405
H	1.0	-2.963877	4.596309	1.310465
C	6.0	3.243581	3.656968	-0.131469
C	6.0	-5.949154	-1.748102	2.388674
H	1.0	-5.841212	-0.712298	2.061930
H	1.0	-6.181979	-1.735811	3.458196
H	1.0	-6.810531	-2.173872	1.873318
C	6.0	-4.979858	-4.024762	2.540328
H	1.0	-5.738942	-4.474339	1.896633
H	1.0	-5.358756	-4.057258	3.567677
H	1.0	-4.091549	-4.655827	2.505328
C	6.0	1.951685	0.246408	-3.135513
H	1.0	1.073328	0.421933	-2.511253
H	1.0	1.685063	0.425283	-4.177964
H	1.0	2.720985	0.961331	-2.846168
C	6.0	-3.824246	1.629577	2.835801
H	1.0	-4.479379	0.787053	2.616913
H	1.0	-4.152742	2.092088	3.766418
H	1.0	-2.807456	1.258918	2.979318
C	6.0	4.501423	-3.716909	1.653984
H	1.0	4.331577	-4.532040	0.952096
H	1.0	4.645516	-4.154375	2.642742

H	1.0	5.417224	-3.192246	1.373885
C	6.0	-4.970387	-2.505148	-2.355117
H	1.0	-4.317025	-1.656017	-2.567142
H	1.0	-5.806518	-2.458565	-3.061274
H	1.0	-4.422150	-3.424197	-2.558493
C	6.0	3.551395	-1.748022	2.845743
H	1.0	4.451155	-1.157633	2.669316
H	1.0	3.666538	-2.284648	3.787455
H	1.0	2.695852	-1.079013	2.944686
C	6.0	2.213882	-4.623873	-1.296145
H	1.0	1.242502	-4.281758	-0.958317
C	6.0	-1.806454	-3.025254	-1.246952
H	1.0	-2.388889	-2.798408	-2.138353
H	1.0	-1.065531	-3.783645	-1.529306
H	1.0	-1.257070	-2.128805	-0.946832
C	6.0	-5.293567	3.191976	1.563156
H	1.0	-5.355924	4.010283	0.846855
H	1.0	-5.630936	3.567383	2.530110
H	1.0	-5.970750	2.394577	1.249111
C	6.0	4.538914	-4.160641	-1.735350
H	1.0	5.372223	-3.468369	-1.718509
C	6.0	-3.147614	-4.968569	-0.419416
H	1.0	-3.772190	-5.391484	0.370234
H	1.0	-2.291722	-5.639805	-0.553723
H	1.0	-3.722455	-4.987247	-1.346035
C	6.0	5.080985	2.275630	2.072707
C	6.0	-5.256387	3.581788	-1.899538
H	1.0	-5.815935	2.654722	-1.935819
C	6.0	-3.207675	1.832781	-3.930679
H	1.0	-4.096951	1.199406	-3.931699
H	1.0	-2.702955	1.720930	-4.891845
H	1.0	-3.516461	2.872664	-3.834524
C	6.0	-1.724544	0.009298	-3.108851
H	1.0	-1.065775	-0.324601	-2.304460
H	1.0	-1.153287	0.015471	-4.038340
H	1.0	-2.543068	-0.702906	-3.210777
B	5.0	0.825254	0.094186	0.695987
B	5.0	-0.731127	0.295525	0.679961
C	6.0	-6.461521	-3.620580	-0.707159
H	1.0	-5.937505	-4.578081	-0.737366
H	1.0	-7.219137	-3.635371	-1.498500

H	1.0	-6.989131	-3.558732	0.244930
C	6.0	3.926127	1.872741	3.009006
H	1.0	3.211538	2.675842	3.167942
H	1.0	4.333157	1.602288	3.988969
H	1.0	3.361005	1.016337	2.640965
C	6.0	2.263152	3.842078	1.037263
H	1.0	1.775456	2.906928	1.322620
H	1.0	1.474463	4.538531	0.730018
H	1.0	2.740995	4.267100	1.919225
C	6.0	3.946789	4.997405	-0.386652
H	1.0	4.593083	5.290285	0.443426
H	1.0	3.193006	5.783300	-0.506608
H	1.0	4.552647	4.987846	-1.293360
C	6.0	5.945251	2.170934	-1.006457
C	6.0	3.637972	-6.343419	-2.187694
H	1.0	3.774398	-7.358809	-2.535799
C	6.0	2.374711	3.304777	-1.347833
H	1.0	2.944446	3.157550	-2.264149
H	1.0	1.669584	4.124337	-1.528790
H	1.0	1.781294	2.408476	-1.152510
C	6.0	2.389998	-5.923603	-1.745898
H	1.0	1.551478	-6.607490	-1.751324
C	6.0	4.710554	-5.461582	-2.184886
H	1.0	5.683247	-5.787253	-2.528780
C	6.0	-3.252317	4.771282	-1.281024
H	1.0	-2.262019	4.771699	-0.843298
C	6.0	7.057544	3.181292	-0.698353
H	1.0	6.684767	4.207215	-0.660088
H	1.0	7.820887	3.137725	-1.483105
H	1.0	7.554344	2.970876	0.248776
C	6.0	-1.070174	2.374124	-2.706933
H	1.0	-1.409635	3.403875	-2.595080
H	1.0	-0.456101	2.320929	-3.608443
H	1.0	-0.451990	2.109267	-1.845483
C	6.0	6.200825	1.239262	2.260044
H	1.0	5.916852	0.256188	1.877996
H	1.0	6.417012	1.131385	3.327767
H	1.0	7.127637	1.536855	1.769279
C	6.0	5.455656	2.433026	-2.438114
H	1.0	4.673410	1.729235	-2.729233
H	1.0	6.291293	2.295388	-3.133042

H	1.0	5.076863	3.444019	-2.580472
C	6.0	6.546842	0.754717	-1.026534
H	1.0	7.025511	0.479937	-0.089264
H	1.0	7.308254	0.700502	-1.812568
H	1.0	5.781817	0.005610	-1.245325
C	6.0	3.711994	-1.392921	-3.802104
H	1.0	4.501388	-0.707897	-3.487517
H	1.0	3.485041	-1.202095	-4.852493
H	1.0	4.077996	-2.415995	-3.716236
C	6.0	5.613633	3.640776	2.527984
H	1.0	6.441553	3.998072	1.912586
H	1.0	5.979028	3.561239	3.557690
H	1.0	4.834287	4.403534	2.521362
C	6.0	-3.791422	5.939587	-1.802057
H	1.0	-3.217534	6.856047	-1.767795
C	6.0	-5.794097	4.752452	-2.409029
H	1.0	-6.784141	4.744302	-2.845161
C	6.0	-5.061321	5.931935	-2.361500
H	1.0	-5.480609	6.845392	-2.762219
C	6.0	1.352006	-2.154737	-3.375760
H	1.0	1.706468	-3.183636	-3.410881
H	1.0	0.996792	-1.889206	-4.373196
H	1.0	0.510757	-2.092676	-2.682259

(26) $B_2(Sil)_2$-$SiCl_2$ initial model M06-2X-D3/Def2-TZVP

Si	14.0	-0.000651	-2.117669	0.001042
Cl	17.0	0.586994	-3.479652	-1.511325
Cl	17.0	-0.589148	-3.478285	1.514285
Si	14.0	-2.455046	0.128450	-0.835696
Si	14.0	2.455124	0.127680	0.835509
Si	14.0	-2.320429	-1.532407	-3.313485
Si	14.0	2.321184	-1.532924	3.313497
O	8.0	-2.941396	-0.373469	-2.308932
O	8.0	2.942086	-0.374316	2.308519
N	7.0	-3.823568	-0.068754	0.348360
N	7.0	3.191540	1.775314	0.521419
N	7.0	3.822802	-0.070373	-0.349398
N	7.0	-3.190568	1.776552	-0.522044
C	6.0	3.950610	1.247809	-0.443843
C	6.0	-3.950524	1.249496	0.442805
C	6.0	4.368228	-1.176879	-1.149036

C	6.0	-4.370530	-1.174958	1.147361
C	6.0	2.703466	3.143128	0.724983
C	6.0	-2.700528	3.143859	-0.724452
C	6.0	3.602440	-1.282853	-2.468693
H	1.0	2.535145	-1.399196	-2.272899
H	1.0	3.946498	-2.152898	-3.030280
H	1.0	3.750710	-0.397213	-3.089614
C	6.0	-3.606176	-1.281530	2.467802
H	1.0	-2.538756	-1.398533	2.273079
H	1.0	-3.951358	-2.151425	3.028932
H	1.0	-3.754516	-0.395888	3.088695
C	6.0	1.845326	3.082500	1.990198
H	1.0	1.013263	2.386694	1.855514
H	1.0	1.436893	4.068521	2.214409
H	1.0	2.443216	2.746463	2.838726
C	6.0	-4.139199	-2.439179	0.318848
H	1.0	-4.661350	-2.368646	-0.637388
H	1.0	-4.509494	-3.310119	0.859597
H	1.0	-3.074087	-2.589328	0.131621
C	6.0	5.869939	-1.006870	-1.381793
H	1.0	6.101873	-0.199448	-2.074705
H	1.0	6.267550	-1.927187	-1.810904
H	1.0	6.384115	-0.821449	-0.437104
C	6.0	4.136972	-2.440982	-0.320325
H	1.0	4.660283	-2.370860	0.635307
H	1.0	4.506007	-3.312162	-0.861549
H	1.0	3.071979	-2.590399	-0.131871
C	6.0	-5.872369	-1.003946	1.378523
H	1.0	-6.104498	-0.196173	2.070963
H	1.0	-6.271026	-1.923888	1.807469
H	1.0	-6.385435	-0.818433	0.433250
C	6.0	-3.854722	4.118146	-0.959257
H	1.0	-4.474685	3.770836	-1.786522
H	1.0	-3.455236	5.100531	-1.215873
H	1.0	-4.487825	4.238921	-0.081436
C	6.0	-1.841550	3.082830	-1.989081
H	1.0	-1.010534	2.385801	-1.854219
H	1.0	-1.431592	4.068431	-2.212373
H	1.0	-2.439280	2.748155	-2.838260
B	5.0	0.735303	-0.387794	0.263086
B	5.0	-0.735780	-0.387689	-0.262280

C	6.0	-1.820774	3.573336	0.453191
H	1.0	-2.390279	3.675061	1.377616
H	1.0	-1.351327	4.536029	0.241580
H	1.0	-1.040174	2.826389	0.612548
C	6.0	3.859163	4.115700	0.959529
H	1.0	4.479239	3.767058	1.786149
H	1.0	3.461219	5.098488	1.216994
H	1.0	4.491804	4.236094	0.081320
C	6.0	1.823462	3.574560	-0.451753
H	1.0	2.392401	3.675945	-1.376560
H	1.0	1.355628	4.537841	-0.239246
H	1.0	1.041605	2.828904	-0.610924
H	1.0	-0.898116	-1.267676	-3.595792
H	1.0	-3.097746	-1.450542	-4.567535
H	1.0	-2.493338	-2.867907	-2.712111
H	1.0	3.099467	-1.451519	4.566980
H	1.0	2.492879	-2.868518	2.711986
H	1.0	0.899237	-1.267366	3.596848
C	6.0	4.761354	1.987992	-1.454697
H	1.0	5.795752	2.042881	-1.110803
H	1.0	4.743566	1.466023	-2.407473
H	1.0	4.388591	2.996571	-1.592472
C	6.0	-4.761371	1.990198	1.453192
H	1.0	-5.795283	2.046783	1.108099
H	1.0	-4.745444	1.467675	2.405686
H	1.0	-4.387342	2.998176	1.591970

(27) $B_2(Sil)_2$-$SiCl_2$ initial model M06-2X-D3/6-311+G(d)

Si	14.0	0.00000000	0.00000000	0.00000000
Cl	17.0	1.63384974	0.00000000	1.37212047
Cl	17.0	-1.63384974	-0.00000000	1.37209371
Si	14.0	-0.11383668	-2.59554051	-2.24210876
Si	14.0	0.11427748	2.59527373	-2.24228764
Si	14.0	2.25568553	-3.40694762	-0.55102052
Si	14.0	-2.25431657	3.40746078	-0.55025806
O	8.0	1.07969815	-3.59390213	-1.72141294
O	8.0	-1.07877408	3.59399275	-1.72116559
N	7.0	-1.72584940	-3.43984282	-2.04909731
N	7.0	0.67950879	3.17468144	-3.89679316
N	7.0	1.72666859	3.43900092	-2.04978211
N	7.0	-0.67952116	-3.17481358	-3.89649731

C	6.0	1.86067083	3.52767357	-3.36996551
C	6.0	-1.86037615	-3.52827806	-3.36922893
C	6.0	2.66552005	3.66254973	-0.93648429
C	6.0	-2.66400733	-3.66432518	-0.93540410
C	6.0	0.31619539	2.77335502	-5.26372083
C	6.0	-0.31729363	-2.77207051	-5.26332163
C	6.0	3.61991593	2.47009619	-0.82347522
H	1.0	3.05453824	1.54295752	-0.70811368
H	1.0	4.26631924	2.58627082	0.04994042
H	1.0	4.25842807	2.38076826	-1.70663406
C	6.0	-3.61931772	-2.47266479	-0.82176876
H	1.0	-3.05461868	-1.54509219	-0.70654811
H	1.0	-4.26518806	-2.58948821	0.05195462
H	1.0	-4.25835412	-2.38370946	-1.70458181
C	6.0	-1.17501693	2.42732229	-5.20014729
H	1.0	-1.35353820	1.60662804	-4.49927750
H	1.0	-1.53407828	2.11786706	-6.18409698
H	1.0	-1.75520624	3.29265276	-4.87194602
C	6.0	-1.79761888	-3.75166973	0.32436597
H	1.0	-1.09363085	-4.58439067	0.24901684
H	1.0	-2.42799960	-3.90394892	1.20234974
H	1.0	-1.23564912	-2.82733242	0.47896492
C	6.0	3.42464869	4.98131870	-1.10790422
H	1.0	4.15752329	4.94993243	-1.91493544
H	1.0	3.96956619	5.20275150	-0.18778653
H	1.0	2.73009099	5.80393478	-1.29516193
C	6.0	1.79977413	3.75046993	0.32368552
H	1.0	1.09652489	4.58385841	0.24879331
H	1.0	2.43067822	3.90199248	1.20142478
H	1.0	1.23702113	2.82663274	0.47837678
C	6.0	-3.42209498	-4.98370759	-1.10668146
H	1.0	-4.15518245	-4.95289353	-1.91354118
H	1.0	-3.96660753	-5.20563929	-0.18644410
H	1.0	-2.72690109	-5.80573346	-1.29415905
C	6.0	-0.51419761	-3.92585513	-6.24983068
H	1.0	0.04045561	-4.80517551	-5.91617274
H	1.0	-0.13885400	-3.63702866	-7.23455485
H	1.0	-1.56111025	-4.20748305	-6.36921551
C	6.0	1.17386834	-2.42568217	-5.20048819
H	1.0	1.35267757	-1.60562695	-4.49893990
H	1.0	1.53211842	-2.11514813	-6.18439548

H	1.0	1.75454356	-3.29117776	-4.87358156
B	5.0	0.02076268	0.78027383	-1.73431682
B	5.0	-0.02070237	-0.78053568	-1.73414738
C	6.0	-1.10194354	-1.52102470	-5.67775500
H	1.0	-2.17164782	-1.71647853	-5.78003725
H	1.0	-0.74051030	-1.14591938	-6.63904653
H	1.0	-0.97003961	-0.73994296	-4.92487934
C	6.0	0.51271388	3.92804173	-6.24925789
H	1.0	-0.04143507	4.80719863	-5.91433593
H	1.0	0.13656448	3.64028724	-7.23398871
H	1.0	1.55962109	4.20947767	-6.36913205
C	6.0	1.10020325	1.52251083	-5.67995032
H	1.0	2.16990494	1.71774166	-5.78267906
H	1.0	0.73806267	1.14853687	-6.64141782
H	1.0	0.96851710	0.74066863	-4.92783586
H	1.0	3.05821985	-2.19782171	-0.81769584
H	1.0	3.11402458	-4.60709391	-0.63801787
H	1.0	1.62601885	-3.33736204	0.78209794
H	1.0	-3.11222252	4.60794778	-0.63684891
H	1.0	-1.62409900	3.33760720	0.78258650
H	1.0	-3.05743361	2.19865688	-0.81661263
C	6.0	3.09748790	3.92054888	-4.11219233
H	1.0	3.14973750	5.01034862	-4.17292163
H	1.0	3.98377852	3.56372786	-3.59201371
H	1.0	3.09585913	3.52080270	-5.12147319
C	6.0	-3.09733676	-3.92146189	-4.11104913
H	1.0	-3.14871766	-5.01125264	-4.17274212
H	1.0	-3.98361658	-3.56582532	-3.59006053
H	1.0	-3.09660273	-3.52082865	-5.11998420

(28)B_2(Sil)$_2$-$SiCl_2$ initial model B3LYP-D3/6-311+G(d)

Si	14.0	-0.000573	-2.197256	0.000933
Cl	17.0	0.561536	-3.554294	-1.566140
Cl	17.0	-0.563604	-3.553083	1.568714
Si	14.0	-2.453567	0.105938	-0.790750
Si	14.0	2.453813	0.105227	0.790668
Si	14.0	-2.545021	-1.428094	-3.459853
Si	14.0	2.546159	-1.428895	3.459647
O	8.0	-3.006340	-0.344960	-2.278781
O	8.0	3.007206	-0.345788	2.278433
N	7.0	-3.836751	-0.078245	0.417020

N	7.0	3.173506	1.775844	0.429544
N	7.0	3.836278	-0.079446	-0.417868
N	7.0	-3.172809	1.776833	-0.429957
C	6.0	3.961378	1.248583	-0.520492
C	6.0	-3.961361	1.249815	0.519675
C	6.0	4.389258	-1.193062	-1.223395
C	6.0	-4.391163	-1.191718	1.221750
C	6.0	2.765163	3.164622	0.709684
C	6.0	-2.762898	3.165382	-0.709006
C	6.0	3.636127	-1.291722	-2.560997
H	1.0	2.568236	-1.434440	-2.388405
H	1.0	4.002112	-2.143508	-3.140601
H	1.0	3.771675	-0.391875	-3.168003
C	6.0	-3.639647	-1.290891	2.560223
H	1.0	-2.571611	-1.434006	2.388848
H	1.0	-4.006641	-2.142640	3.139243
H	1.0	-3.775546	-0.391114	3.167248
C	6.0	1.875987	3.093906	1.963252
H	1.0	1.000952	2.462263	1.788877
H	1.0	1.524255	4.091945	2.234985
H	1.0	2.429895	2.681423	2.809572
C	6.0	-4.154067	-2.471409	0.403219
H	1.0	-4.665738	-2.416450	-0.560984
H	1.0	-4.534593	-3.338727	0.946820
H	1.0	-3.090792	-2.639159	0.226731
C	6.0	5.902437	-1.025240	-1.441263
H	1.0	6.149103	-0.205875	-2.117464
H	1.0	6.307811	-1.937635	-1.885079
H	1.0	6.414962	-0.857550	-0.490289
C	6.0	4.152564	-2.472729	-0.404713
H	1.0	4.665442	-2.418100	0.558870
H	1.0	4.532002	-3.340176	-0.948868
H	1.0	3.089430	-2.640003	-0.226943
C	6.0	-5.904525	-1.023219	1.437819
H	1.0	-6.151633	-0.203640	2.113601
H	1.0	-6.310830	-1.935374	1.881280
H	1.0	-6.415843	-0.855426	0.486215
C	6.0	-3.984275	4.046725	-1.019150
H	1.0	-4.566159	3.616268	-1.837445
H	1.0	-3.658472	5.045927	-1.320699
H	1.0	-4.645813	4.165745	-0.159544

C	6.0	-1.873151	3.094533	-1.962162
H	1.0	-0.998810	2.461953	-1.787698
H	1.0	-1.520326	4.092391	-2.233146
H	1.0	-2.427028	2.683064	-2.808998
B	5.0	0.740416	-0.462766	0.253518
B	5.0	-0.740621	-0.462539	-0.252734
C	6.0	-1.936834	3.735127	0.459097
H	1.0	-2.527551	3.842921	1.371312
H	1.0	-1.543547	4.723092	0.203619
H	1.0	-1.095913	3.075644	0.679915
C	6.0	3.987630	4.044483	1.019762
H	1.0	4.569466	3.612894	1.837493
H	1.0	3.663048	5.043846	1.322094
H	1.0	4.648860	4.163324	0.159891
C	6.0	1.939104	3.735998	-0.457624
H	1.0	2.529438	3.843693	-1.370096
H	1.0	1.547071	4.724249	-0.201318
H	1.0	1.097310	3.077616	-0.678386
H	1.0	-1.140918	-1.192864	-3.865545
H	1.0	-3.443067	-1.185750	-4.616383
H	1.0	-2.720428	-2.814758	-2.973808
H	1.0	3.445278	-1.187322	4.615506
H	1.0	2.720320	-2.815571	2.973194
H	1.0	1.142529	-1.192864	3.866505
C	6.0	4.789836	1.993317	-1.519785
H	1.0	5.829353	2.028386	-1.184191
H	1.0	4.766448	1.495122	-2.487150
H	1.0	4.440214	3.013392	-1.645654
C	6.0	-4.790034	1.994834	1.518575
H	1.0	-5.829127	2.031408	1.181817
H	1.0	-4.768333	1.495982	2.485628
H	1.0	-4.439328	3.014411	1.645506

(29) $B_2(Sil)_2$-$SiCl_2$ final model M06-2X-D3/6-311+G(d)

Si	14.0	-0.000000	0.000000	0.000000
Cl	17.0	1.633850	1.372120	0.000000
Cl	17.0	-1.633850	1.372094	-0.000000
Si	14.0	-0.113837	-2.242109	-2.595541
Si	14.0	0.114277	-2.242288	2.595274
O	8.0	1.079698	-1.721413	-3.593902
O	8.0	-1.078774	-1.721166	3.593993

N	7.0	-1.725849	-2.049097	-3.439843
N	7.0	0.679509	-3.896793	3.174681
N	7.0	1.726669	-2.049782	3.439001
N	7.0	-0.679521	-3.896497	-3.174814
C	6.0	1.860671	-3.369966	3.527674
C	6.0	-1.860376	-3.369229	-3.528278
B	5.0	0.020763	-1.734317	0.780274
B	5.0	-0.020702	-1.734147	-0.780536
H	1.0	-2.490202	-1.394026	-3.487014
H	1.0	-0.510020	-4.882696	-3.067387
H	1.0	-2.763147	-3.915017	-3.785912
H	1.0	1.698013	-1.091131	-3.211846
H	1.0	-1.696898	-1.090567	3.212153
H	1.0	2.491328	-1.395035	3.485708
H	1.0	2.763288	-3.916075	3.785164
H	1.0	0.509489	-4.882948	3.067672

(30) CuCl-SiH_2 M06-2X-D3/6-311+G(d)

Si	14.0	0.00008881	-2.28533561	0.00000000
H	1.0	1.14045380	-3.24393275	0.00000000
H	1.0	-1.14026387	-3.24393902	-0.00000000
Cu	29.0	0.00000000	0.00000000	0.00000000
Cl	17.0	0.00000000	2.15397063	0.00000000

(31) CuCl-$SiCl_2$ M06-2X-D3/6-311+G(d)

Si	14.0	0.00217535	-2.30098304	0.00000000
Cl	17.0	1.61970261	-3.53507377	0.00000000
Cl	17.0	-1.61353805	-3.53813765	-0.00000000
Cu	29.0	0.00000000	0.00000000	0.00000000
Cl	17.0	0.00000000	2.14547451	0.00000000

(32) AgCl-SiH_2 M06-2X-D3/6-311+G(d)

Si	14.0	0.00022633	-2.42651595	0.00000000
H	1.0	-1.13759842	-3.38750200	-0.00000000
H	1.0	1.13924291	-3.38611244	0.00000000
Ag	47.0	0.00000000	0.00000000	0.00000000
Cl	17.0	0.00000000	2.36996119	0.00000000

(33) AgCl-$SiCl_2$ M06-2X-D3/6-311+G(d)

Si	14.0	0.00047985	-2.45535196	0.00000000
Cl	17.0	1.61714758	-3.69344945	0.00000000

Cl	17.0	-1.61568318	-3.69478643	-0.00000000
Ag	47.0	0.00000000	0.00000000	0.00000000
Cl	17.0	0.00000000	2.36117608	0.00000000

(34) AuCl-SiH_2 M06-2X-D3/6-311+G(d)

Si	14.0	0.00106669	-2.20631157	0.00000000
H	1.0	1.16546544	-3.12460141	0.00000000
H	1.0	-1.16050989	-3.12809858	-0.00000000
Au	79.0	0.00000000	0.00000000	0.00000000
Cl	17.0	0.00000000	2.34352512	0.00000000

(35) AuCl-$SiCl_2$ M06-2X-D3/6-311+G(d)

Si	14.0	0.00016945	-2.20688099	0.00000000
Cl	17.0	1.61449719	-3.43134668	0.00000000
Cl	17.0	-1.61439880	-3.43161931	-0.00000000
Au	79.0	0.00000000	0.00000000	0.00000000
Cl	17.0	0.00000000	2.32875400	0.00000000

(36) CoCl(C_6H_6)-SiH_2 M06-2X-D3/6-311+G(d)

Si	14.0	0.00000000	0.00000000	0.00000000
H	1.0	0.00000000	1.48803163	0.00000000
H	1.0	1.48487531	-0.26184548	0.00000000
Co	27.0	-1.69707796	-1.37330293	0.00003394
Cl	17.0	-3.09201009	0.40262655	0.00097742
C	6.0	-0.87988443	-3.37670485	0.70099405
C	6.0	-2.07158913	-3.16670737	1.41566327
C	6.0	-3.23721135	-2.86785371	0.70653137
C	6.0	-3.23829949	-2.86720505	-0.70520726
C	6.0	-2.07380554	-3.16545304	-1.41645599
C	6.0	-0.88098127	-3.37613550	-0.70386460
H	1.0	0.05128739	-3.52562810	1.23301668
H	1.0	-2.06912856	-3.16181766	2.49783313
H	1.0	-4.13843441	-2.58582059	1.23508869
H	1.0	-4.14032356	-2.58462717	-1.23210465
H	1.0	-2.07306671	-3.15958289	-2.49862256
H	1.0	0.04934314	-3.52465488	-1.23747870
